# Performance Analysis of Optically Pumped ^4^He Magnetometers vs. Conventional SQUIDs: From Adult to Infant Head Models

**DOI:** 10.3390/s22083093

**Published:** 2022-04-18

**Authors:** Saeed Zahran, Mahdi Mahmoudzadeh, Fabrice Wallois, Nacim Betrouni, Philippe Derambure, Matthieu Le Prado, Agustin Palacios-Laloy, Etienne Labyt

**Affiliations:** 1 INSERM, U1105, GRAMFC, Université de Picardie Jules Verne, CHU Sud, 80000 Amiens, France; saeedzahran@hotmail.com (S.Z.); mahdi.mahmoudzadeh@u-picardie.fr (M.M.); fabrice.wallois@u-picardie.fr (F.W.); 2 INSERM, U1172, CHU de Lille, Université de Lille, Degenerative & Vascular Cognitive Disorders, 59000 Lille, France; nacim.betrouni@inserm.fr (N.B.); philippe.derambure@chru-lille.fr (P.D.); 3 Laboratoire d’Electronique et de Technologies de l’Information, CEA, 38054 Grenoble, France; matthieu.leprado@cea.fr (M.L.P.); agustin.palacioslaloy@cea.fr (A.P.-L.); 4 Mag4health, 9 Avenue Paul Verlaine, 38000 Grenoble, France; 5 CEA Tech Hauts de France, 59000 Lille, France

**Keywords:** optically pumped ^4^He magnetometer, SQUID, forward and inverse models

## Abstract

Optically pumped magnetometers (OPMs) are new, room-temperature alternatives to superconducting quantum interference devices (SQUIDs) for measuring the brain’s magnetic fields. The most used OPM in MagnetoEncephaloGraphy (MEG) are based on alkali atoms operating in the spin-exchange relaxation-free (SERF) regime. These sensors do not require cooling but have to be heated. Another kind of OPM, based on the parametric resonance of ^4^He atoms are operated at room temperature, suppressing the heat dissipation issue. They also have an advantageous bandwidth and dynamic range more suitable for MEG recordings. We quantitatively assessed the improvement (relative to a SQUID magnetometers array) in recording the magnetic field with a wearable ^4^He OPM-MEG system through data simulations. The OPM array and magnetoencephalography forward models were based on anatomical MRI data from an adult, a nine-year-old child, and 10 infants aged between one month and two years. Our simulations showed that a ^4^He OPMs array offers markedly better spatial specificity than a SQUID magnetometers array in various key performance areas (e.g., signal power, information content, and spatial resolution). Our results are also discussed regarding previous simulation results obtained for alkali OPM.

## 1. Introduction

Magnetoencephalography (MEG) enables the non-invasive characterization of human brain function and thus provides unique insights into the neural substrates that underpin functions and dysfunctions processes in both research [1,2] and clinical [3,4] settings. Although SQUID-based MEG has a high spatial resolution (by minimizing the volume conduction effect and amplitude attenuation through skull conductivity), the technique has some inherent limitations. Firstly, liquid helium (He) must be used to reach sufficiently low temperatures. Secondly, the cryogenic constraint limits the flexibility of sensor configurations, and a SQUID-based system cannot be adjusted to suit different individual head shapes and sizes (e.g., as encountered with children and infants) [5]. Baby MEG systems have been developed [6], however they are not widely available. Thirdly, systems tailored to a baby’s head size are still limited by their rigid sensor configuration and the thermal insulation required by the cryogenic sensors; a distance of at least about three cm between the sensors and the subject’s scalp is typically required. Reducing this distance as much as possible is mandatory as the amplitude of the magnetic field decreases with the cube of the sensor-scalp distance *r* (i.e., vs. $\frac{1}{r^{3}}$). Fourthly, head movements inside the fixed cap (support + sensors) also impede the accuracy of source localization, since the sensors will not always be recording the same regions of the brain. This might be a limitation in a patient who makes uncontrolled movements (e.g., a patient with Parkinson’s disease) [5] and in children and infants. Lastly, the size of SQUID-based MEG systems also necessarily requires the use of large, costly, heavy magnetically shielded rooms (MSRs) and this imposes strong infrastructural constraints on buildings, in which these devices are installed. Overall, the ability to capture brain function via a “wearable” scanning device (i.e., one that can be adapted to fit any individual subject’s head shape and size, and that enables free movement during recording) is of major interest.

Currently, electroencephalography (EEG) systems are the most widely used wearable neuroimaging devices. EEG instrumentation is lightweight and flexible and can be adapted to any head size—making it usable in almost any experimental setting. However, EEG has significant technological drawbacks, relative to MEG. The head’s inhomogeneous conductivity profile reduces the amplitude of the electrical potentials and spatially blurs the signal on the scalp surface [7,8]. These factors place a practical limit on the spatial resolution of EEG [9]. Accordingly, wearable MEG (giving a higher spatial resolution) is a compelling prospect.

The recent development of new MEG sensors (such as alkali optically pumped magnetometers [OPMs] [10]) has now produced the sensitivity gains required for the detection of neuromagnetic fields. OPMs working in the spin-exchange relaxation-free (SERF) regime demonstrated a sensitivity of 5 fT/$\sqrt{\mathrm{Hz}}$ with a 6 × 6 ${\mathrm{cm}}^{2}$ footprint [11]. Mhaskar and colleagues [12] created a smaller chip-scale microfabricated OPM with a volume of around 1 ${\mathrm{cm}}^{3}$ and a sensitivity of more than 20 fT/$\sqrt{\mathrm{Hz}}$. An OPM with a volume of 2 × 2 × 5 ${\mathrm{cm}}^{3}$ and a sensitivity of 10 fT/$\sqrt{\mathrm{Hz}}$ was demonstrated by Shah and Wakai [13]. The advantages of being able to place these new MEG imaging sensors closer to the brain have already been demonstrated in adults [14,15]. By eliminating the need for liquid He, the drastic reduction in the system’s size and brain-to-sensor distance has enabled the implementation of a wearable MEG headset [16]. Removing the Dewar containing liquid, He also reduces the size of the MEG system, and thus, the cost of the MSR. However, these alkali OPMs suffer from a small dynamic range and a narrow bandwidth. The small dynamic range requires perfect control of the ambient magnetic field inside the MSR, by using field-nulling coil systems [5,17,18] to limit the saturation of these sensors as soon as the magnetic field offset exceeds 5 nT. Limiting the bandwidth to 100 Hz prevents the recording of fast brain oscillations, such as high gamma band activity. Furthermore, alkali OPMs have to be heated to over 100 °C. Dissipation of this heat requires the sensor to be moved a few millimeters away from the scalp, which impedes the recording of brain activity. These alkali OPMs are also limited to one or two axes measurement of the brain’s magnetic field.

Some of the limitations of alkali OPMs can be overcome by using OPMs based on helium four atoms [19] in their *F* = one metastable level, which can be significantly populated by using a low-intensity radio-frequency discharge of only a few milliwatts. Since helium is a gas at room temperature, these ^4^He OPMs can be operated at room temperature (i.e., with no need for heating or cooling) and can be placed in direct contact with the subject’s scalp (thus optimally reducing the brain-sensor distance). They have a large dynamic range (up to 200 nT) and a broad bandwidth (ranging from direct current to two kHz); these features enable the full spectrum of normal and pathological brain activities to be recorded. Furthermore, ^4^He OPMs operate more easily in a noisier magnetic environment. These devices provide exhaustive, three-axis measurements of the brain’s magnetic field thanks to two orthogonal rotating RF fields at different frequencies to record the double parametric resonance of He atoms. The concept of MEG recording with these ^4^He OPMs has been proved [20]. The amplitude of brain responses is higher for ^4^He OPMs than for SQUIDs, thanks to the sensor’s proximity to the scalp. Since the proof-of-concept study, the sensitivity of ^4^He OPM systems has increased five-fold. A detailed technical description of ^4^He OPMs is reported in [21,22].

The primary objective of the present study was to assess the added value of a ^4^He OPM, relative to SQUIDs and alkali OPMs. To this end, a ^4^He OPM sensor array’s performance variables were modeled and compared with those obtained for an equivalent SQUID MEG array and a state-of-the-art alkali OPM array. The study’s secondary objective was to assess the added value of a ^4^He OPM sensor as a function of the subject’s age. To this end, the same variables were computed for the geometries of 12 healthy subjects, including adults, children, and infants. For SQUID MEG simulations, two different configurations were used: (i) a head-sized SQUID array (as used in a baby MEG system—the optimal set-up, yet one which is only available in a few centers worldwide); and (ii) a head position translated along the vertical axis, as more usually performed with standard SQUID MEG systems (where the top of the baby’s head is placed close to the MEG helmet). The metrics derived from forward and inverse models included the signal power, information capacity, localization accuracy, and the dependency of the respective MEG and OPM signal magnitudes on the orientation of a current dipole.

## 2. Materials and Methods

### 2.1. Head Models

T1-weighted MRI datasets were obtained from 12 healthy subjects aged from one month to the adult age. In each case, the brain, skull and scalp compartments were segmented using FreeSurfer software [23]. A surface mesh of the cortical gray-white matter border was created with roughly 300,000 vertices, which was subsequently downsampled to 15,002 vertices. Skull and scalp surfaces were triangulated and decimated to obtain meshes with 1922 vertices each. The boundary element method (BEM [24]) was used to create the head model. We assumed that the brain:skull:scalp conductivity ratio was 1:0.0125:1 [25].

### 2.2. Source Space for the Cortex Surface and the Brain Volume

First, 2D topographic maps related to a single dipole simulated over the somatosensory area (the post central gyrus, hand area) with various orientations (one near-radial and two tangential) were computed, in order to characterize the magnetic field mapping along the ^4^He OPM’s three measurement axes for various dipolar brain source orientations. To enable more flexible simulation of the sources in various dimensions (such as depth and orientation), the source space was built within the brain volume. We used a source grid with 12,321 isotropic points within the brain volume and a grid resolution of 5 mm, and considered three orthogonal orientations per dipole on the x-, y-, and z-axes with unconstrained sources. The characterization of all the dipoles yielded a 12,321 × 3 matrix.

For all other simulations of the brain’s magnetic field and the computation of quantitative metrics, the source space was computed from the cortex surface mesh. Each vertex of the brain mesh was used to set a dipole source’s position, leading to a source space of 15,002 dipoles. The primary current distributions were considered to be at a depth of five mm in the cortical mantle.

### 2.3. Sensor Models

The sensor geometries are described in Table 1. In total, four integration points were uniformly distributed throughout the volume of the ^4^He OPM sensor (a cube with a side length of 10 mm). The standard SQUID sensor model implemented in the Brainstorm software was used in the present study [26]. The noise level was set to 40 fT$\sqrt{\mathrm{Hz}}\;$for ^4^He OPMs (as recommended in [21]), and 5 fT$\sqrt{\mathrm{Hz}}$ for SQUID magnetometers. With regard to the distances *d*, the distance between the bottom part of the He gas cell and the scalp was considered as this was previously used in simulation work comparing alkali OPM [15]. This distance was reasonably set to three mm, while the distance between SQUID and the scalp was set to 2 cm as previously used in [15].

Two different configurations were used for SQUID MEG simulations in infants and children: (i) mSQUID (optimized), i.e., a baby MEG device for which the sensor-scalp distance (2 cm) is optimized all around the head, and (ii) mSQUID (standard), corresponding to a conventional SQUID MEG system, for which the top of the infant’s head was placed as close as possible to the helmet (around 2 cm from the sensors, along the z axis). Given the size of the adult head model, only the mSQUID (standard) configuration was considered (as usually performed during MEG examinations). The mSQUID sensor array still comprised N = 102 MEG magnetometers, which measured the magnetic field component that was nearly radial to the scalp’s surface.

Four OPM arrays were developed: three 102-sensor arrays that, respectively, measured the normal component of the magnetic field (OPMn), the first orthogonal tangential component (OPMt_1_) and the second orthogonal tangential component (OPMt_2_), and a 306-sensor combination of the three arrays that measured all field components (OPMa). The OPM sensor (the center of the ^4^He gas cell) was 3 mm away from the scalp.

**Figure 1:**
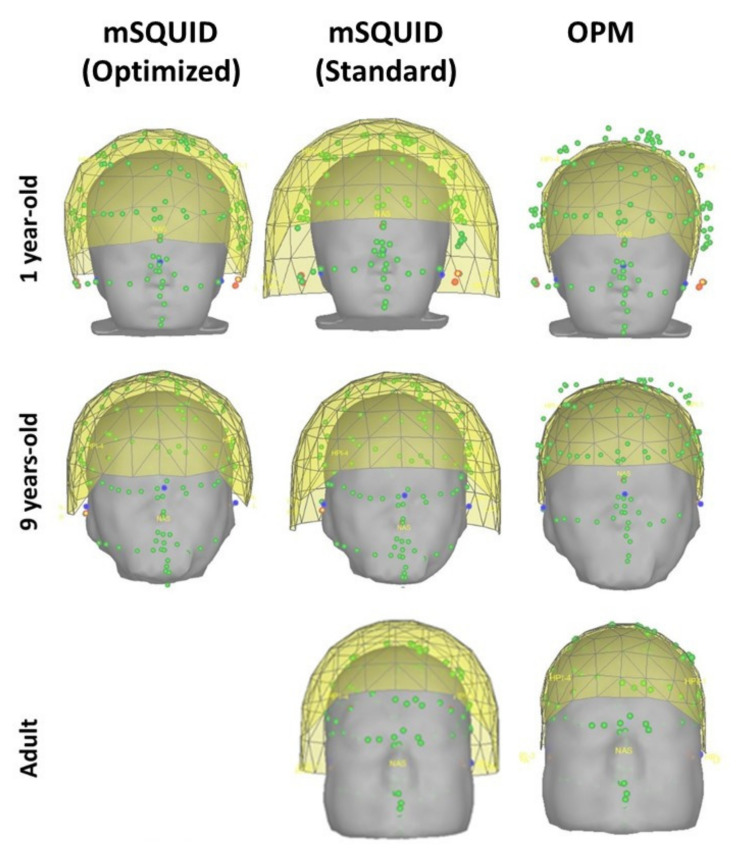
The frontal views of the mSQUID (Optimized) (**left**), mSQUID (Standard) (**middle**) and OPM (**right**) sensors helmet for three different subjects (a 1-year-old, a 9-year-old, and an adult).

Figure 1 represents the mSQUID (optimized), mSQUID (standard), and OPM sensor locations at different ages (an adult, a 9-year-old, and a 1-year-old). As mentioned above, only the mSQUID (standard) array was built for the adult.

### 2.4. The Forward Model

The forward problem occurs in computing, from a given electrical source (dipole), the electrical potential (or its related magnetic field obtained by integrating the total current using Biot–Savart law) at the sensors level.

MEG and OPM signals *S* ∈ ${\mathbb{R}}^{MXT}$ recorded from *M* channels for *T* time samples can be described as the weighted sum of the dipole signals *D* ∈ ${\mathbb{R}}^{dXT}$.

(1) S=L·D+N

where *L* ∈ ${\mathbb{R}}^{MX3d}$ are the matrices containing the dipolar source lead fields that link the MEG or OPM signals in an array of *M* sensors to the three components of a dipole moment vector at locations in the brain. *N* is the additive noise. The forward matrix was computed using OpenMEEG software [24].

### 2.5. The Inverse Model

Given the MEG signals *S*(*t*) and the gain matrix *L*, the inverse problem consists of finding an estimate *D*(*t*) of the dipolar source parameters. We used minimum norm estimates [25,27] (based on the L2 norm) to regularize the problem and search for the solution with the minimum power. This form of estimator is best suited to distributed source models, in which the dipole activity is expected to span some portions of the cortical surface:

(2) $$D_{MNE}=L^{T}{\left(LL^{T}+\lambda C\right)}^{-1}$$

where λ is the regularization parameter, and *C* is the noise covariance matrix. The (symmetric) resolution matrix in this case is:

(3) $$R_{MNE}=L^{T}{\left(LL^{T}+\lambda C\right)}^{-1}L\;$$

For linear estimators, the resolution matrix is a valuable tool for describing the spatial resolution [28]. The relationship between the estimated and modeled current distributions is represented by the resolution matrix *R*.

### 2.6. Evaluation Criteria

#### 2.6.1. Signal Power

The $L_{2}$-norm of the source topography squared was used to define the topography power of the *i*th source:

(4) $$SP=\|t_{i}\|{}^{2}$$

where *t_i_* = (*C*^−1/2^ *L*)*_i_* is the whitened topography of the *i*th source.

If we assume that all sensors in the array have an equal sensor noise variance *σ*^2^, the topography power is linearly proportional to the source’s signal-to-noise ratio.

#### 2.6.2. Contributions of Primary and Volume Currents

The contributions of the primary and volume currents to the total magnetic field were also investigated for the various sensor arrays. The ratio between the total current and the primary current was defined as TP:

(5) $${\mathrm{TP}}_{i}=\frac{\left\|p_{i}\;+\;v_{i}\right\|}{\left\|p_{i}\right\|}=\frac{\left\|t_{i}\right\|}{\left\|p_{i}\right\|}$$

where the *i*th columns of the corresponding matrices *P V*, *L* are denoted by $p_{v}$, $i_{i}$ and $t_{i}$, *P* is the primary current, *V* is the volume current, and *L* = *P* + *V* is the total current.

By computing the ratio between the field components’ norms, we determined the relative overall magnitude of the primary and volume currents’ topographies for the various sensor arrays.

(6) $${\mathrm{PV}}_{i}=\frac{\left\|p_{i}\right\|}{\left\|v_{i}\right\|}$$

#### 2.6.3. Total Information

$I_{tot}$ (the information per sample of the multi-channel system) uses a single number to quantify all aspects of forward-model-based metrics [29,30], according to Shannon’s theory of communication [31]:

(7) $$I_{tot}=\frac{1}{2}\overset{N_{c}}{\underset{i=1}{\sum }}log_{2}\left(SNR_{i}^{ch}+1\right)$$

where $SNR_{i}^{ch}\;$is the signal-to-noise ratio of the *i*th channel.

#### 2.6.4. Singular Value Decomposition (SVD)

We generated the singular value decomposition of the *N* × 3-dimensional dipolar gain matrix at each location indexed by *k* = 1,…, *M* to investigate the sensitivity of the mSQUID and OPM sensor arrays to sources with varied orientations [32]:

(8) $$A_{k}=\overset{3}{\underset{i=1}{\sum }}\lambda_{k,j}u_{k,j}v_{k,j}^{T}$$

where $u_{k,j}\;$and $v_{k,j}^{\;}\;$are the left and right singular vectors, respectively, and the singular values are $\lambda_{k,1,}$, $\lambda_{k,2}$ and $\lambda_{k,3}$. We denoted the largest and smallest singular values (corresponding to the dipole orientations, to which the mSQUID or OPM sensor array is the most and the least sensitive) as $\lambda_{k,1}$ = $\lambda_{k,max}$ and $\lambda_{k,1}$ = $\lambda_{k,min}$, respectively. $\lambda_{k,max}$ corresponds to the tangentially oriented brain sources, to which MEG is most sensitive, whereas $\lambda_{k,min}$, corresponds to the radially oriented brain sources, to which MEG is least sensitive.

#### 2.6.5. Dipole Localization Error

From the inverse model and the resolution matrix *R* in particular, it is possible to compute various metrics to assess the performance of the source localization solution. Notably, the point-spread functions (PSFs) describe how an imaging system distorts a point source. The *i*th column of the resolution matrix *R* corresponds to the PSF for the source *i*:

(9) $${\mathrm{PSF}}_{i}=R_{i}$$

To assess the performance of the source imaging results obtained in our mSQUID and OPM data simulations, and to characterize the similarity between the original and the estimated source configurations, we used a PSF-derived metric: the dipole localization error (DLE) [33]:

(10) $$\sqrt{\left(r_{s}^{i}-r_{p}^{i}\right)}$$

where $r_{s}^{i}$ is the true location of the source *i* and $r_{p}^{i}$ is the location of the PSF peak for the source *i*. Therefore, DLE quantifies the distance between the original and estimated source locations

#### 2.6.6. Spatial Dispersion

This index (developed by Molins et al. [34]) quantifies the spatial dispersion (SD) of the estimated source distribution around the true source location:

(11) $$\sqrt{\frac{\sum_{j=1}^{N_{s}}{\left(a_{j}\left(r_{i}^{p}-r_{i}\right)\right)}^{2}}{\sum_{j=1}^{N_{s}}{\left(a_{j}\right)}^{2}}}$$

where $N_{s}$ is the number of sources, $a_{j}$ is the intensity of the source *j*, and $r_{j}$ is the location of the source *j*.

### 2.7. Statistical Analysis

For each of the metrics, a t-test was used to determine whether or not there was a significant difference between ^4^He OPM and mSQUID (standard) or between ^4^He OPM and mSQUID (optimized) when this configuration was used for data simulation (children and infants). Pairwise, comparison with a t-test has been used as the simulated data for He OPM and SQUID were generated with the same headmodel for a given age.

Furthermore, a one-way analysis of variance (ANOVA) was also used to determine whether or not there was a significant age effect on the different metrics. When a significant effect of age was revealed, *post hoc* analyses were used to determine which specific ages differed from each other.

### 2.8. Data Simulation Procedure and Metric Computation for the Comparative Analysis

**Figure 2:**
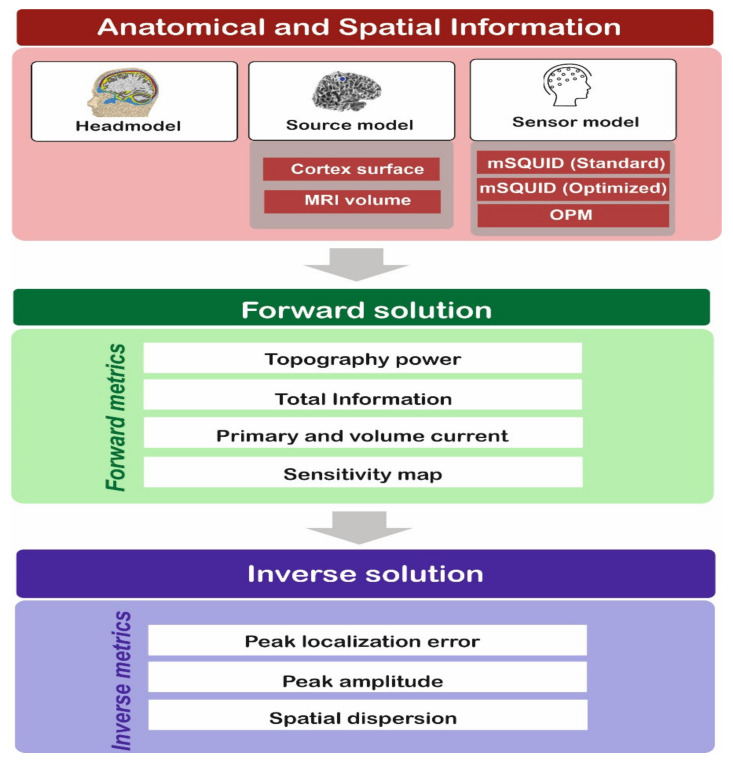
The steps in the comparative analysis.

The steps in the comparative analysis are summarized in Figure 2. In step 1, structural MR images were processed using the automated segmentation algorithms in FreeSurfer software [23]. A boundary element model (BEM) was created using a watershed algorithm. In step 2, the lead field matrix *L* was estimated using the boundary element model and all the metrics related to the forward solution were computed. In step 3, the temporal dynamics of dipolar sources *D*(*t*) were estimated from the scalp MEG and OPM signals *S*(*t*). The L2-norm MNE (minimum norm estimate) was used to estimate *D*(*t*). The noise covariance matrices were computed for each sensor and each subject. The default signal-to-noise ratio (=3) in the Brainstorm software was used for regularization. The metrics related to the resolution matrix were also computed, so that the performance of each sensor could be assessed. All the simulations described here were executed using the Brainstorm toolbox [26].

## 3. Results

### 3.1. Topographic Mapping

**Figure 3:**
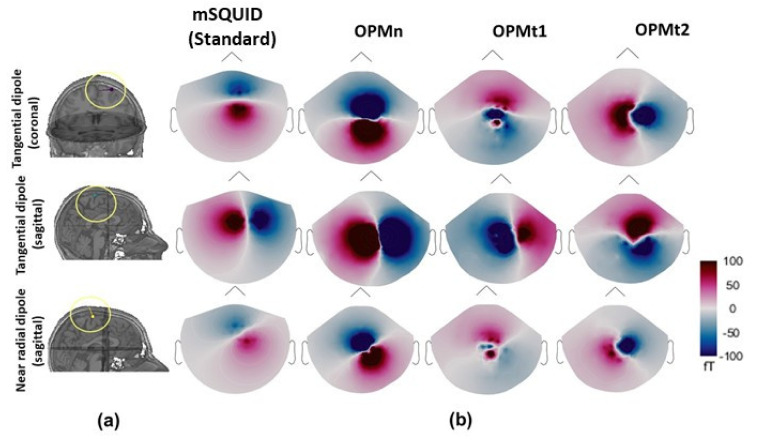
(**a**) The position of the dipoles with radial and tangential orientations in the somatosensory region of an adult subject. (**b**) Topographic maps of the magnetic field components of the radial and tangential dipoles for mSQUID, OPMn, OPMt1, and OPMt_2_. The shape of the 2D map differed from one type of sensor array to another.

A typical 2-dimensional (2D) map of the magnetic field distributions in the adult subject is shown in Figure 3. For the different sensor setups, a qualitative visual inspection of the identified topography map showed that results were highly dependent on the type of sensor used. mSQUID and OPMn gave similar topographies, however, the magnetic field amplitude recorded with the OPMn array was larger. OPMt_1_ and OPMt_2_ provided orthogonal maps, as expected, and additional information with respect to OPMn. Interestingly, the magnetic field generated by the near-radial dipole was recorded by OPMt_1_ and OPMt2. These results were based on a single dipole and so were only qualitative. Our quantitative assessment of the benefit of ^4^He OPM vs. mSQUID with a full source space is described in the following sections. Furthermore, as previously detailed in the methods section, these quantitative analyses have been performed with different headmodels corresponding to different ages as the head size is an important factor in the performance assessment of MEG sensors arrays. All the results will be reported with respect to age in the next sections.

### 3.2. Forward Metrics

#### 3.2.1. Topographic Power

**Figure 4:**
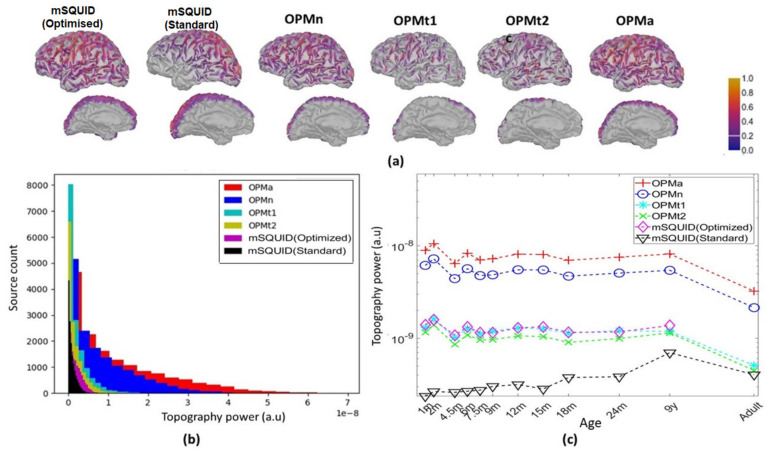
(**a**) Topographic powers of the arrays (for the nine-year-old subject). (**b**) Histograms of the distribution of the topographic power for sources in both hemispheres, for the same subject. (**c**) The topographic powers for various configurations of the sensor arrays, by subject age.

The topographic powers of the different sensor array configurations for the nine-year-old subject are shown in Figure 4a. The corresponding histogram is shown in Figure 4b, and the topographic power as a function of age is shown in Figure 4c. With the adult subject, the topographic power was 8.9 times greater for OPMa than for mSQUID and, respectively, 3.6 and 4.1 times greater for OPMn than for the OPMt_1_ and OPMt_2_.

#### 3.2.2. Total Information

**Figure 5:**
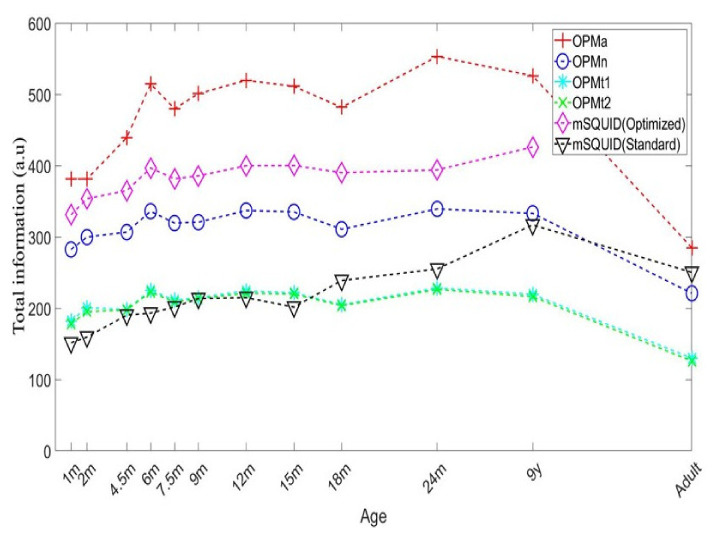
The total information capacities for the sensor arrays, by subject age.

The total information capacities of the different sensor configurations are shown in Figure 5. For the adult, combining the OPM arrays (OPMa) increased the information capacity 1.137-fold, relative to mSQUID. The mSQUID array increased the information capacity 1.133-fold (relative to OPMn) and 1.9-fold (relative to OPMt_1_ and OPMt_2_).

#### 3.2.3. Sensitivity Map

**Figure 6:**
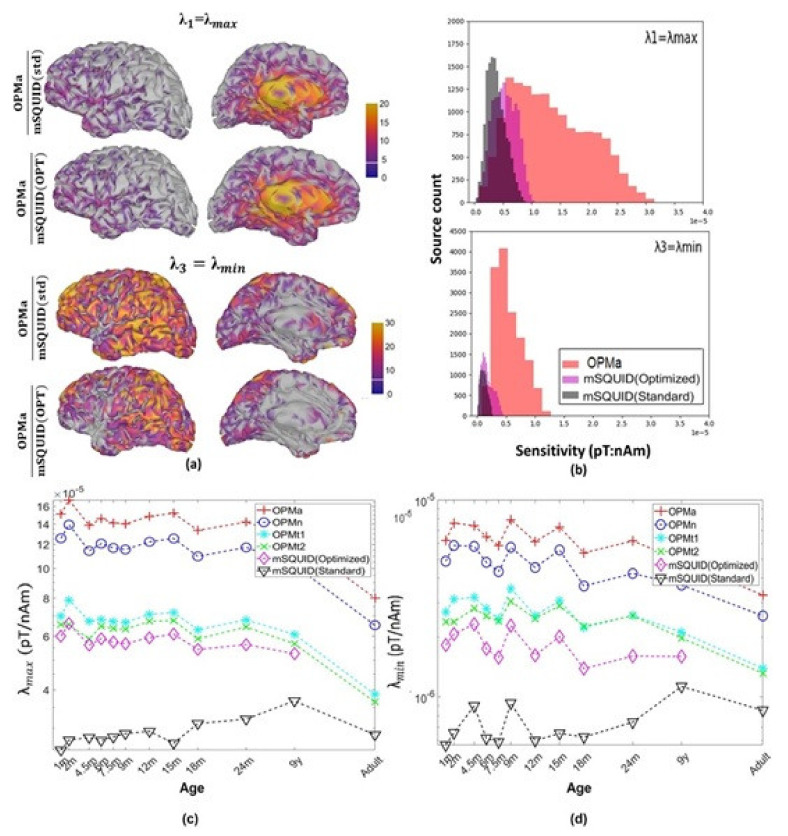
(**a**) The relative sensitivity of the arrays to the orthogonal source components at each location on the cortex, as indicated by the singular values of the dipole gain matrix for the nine-year-old subject. (**b**) Histograms for $\lambda_{min}$ and $\lambda_{max}$ at all locations on the cortex, for the same subject. (**c**,**d**) Distribution of the sensitivity for $\lambda_{max}$ and $\lambda_{min}$, by subject age.

For OPMa and mSQUID, the distributions of the relative sensitivity of the orthogonal source orientations across the cerebral cortex are shown in Figure 6a. The histograms of $\lambda_{min}$ (radially oriented brain sources) and $\lambda_{max}\;$(tangentially oriented brain sources) for the nine-year-old subject are shown in Figure 6b. The distributions of the sensitivity for all the subjects are given in Figure 6c,d. The prominence of low $\lambda_{min}$ values for mSQUID (Figure 6b) show that for most places on the cortex, there was a source orientation, at which low or no MEG signals were created. The maps (Figure 6a) and histograms (Figure 6b) show that the sensitivity for source orientations corresponding to $\lambda_{min}\;$and to $\lambda_{max}$ was higher for OPMa than for mSQUID. With the adult subject, $\lambda_{min}\;$and $\lambda_{max}\;$were, respectively, 2.98 and 3.71 times greater for OPMa than for mSQUID.

#### 3.2.4. Primary Current and Volume Current Contributions

**Figure 7:**
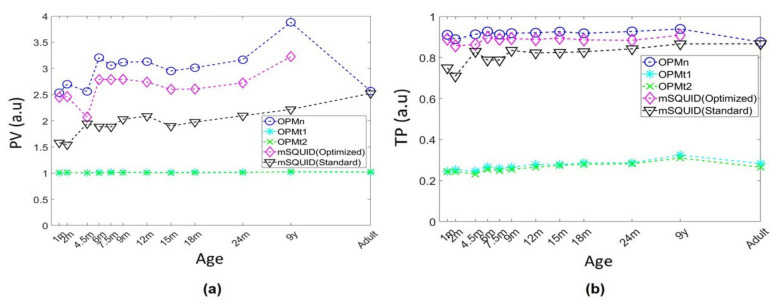
(**a**) The norm ratios between primary and volume current topographies (PV) and (**b**) between total and primary current topographies (TP) for all sensors, by subject age.

The topographies’ primary current and volume current components are shown in Figure 7. Since the values of PV are close to one for OPMt_1_ and OPMt_2_, the total amplitudes of the volume current topographies are similar to those of the primary current. Since TP was well below one for OPMt_1_ and OPMt_2_ (mean value: 0.25–0.30), the magnetic field induced by the volume currents significantly reduced the overall amplitude of the primary-current topography in tangential measurements. The total magnitude of the primary current topography was greater than that of the volume currents for OPMn and mSQUID, since PV was much greater than one. Since TP was close to one, the volume currents did not result in a significant decrease in the overall amplitude of the primary current topography in OPMn and mSQUID arrays. With the adult head model, the PV and TP of the OPMn were, respectively, 1.03- and 1.005-fold greater than those of the mSQUID.

### 3.3. Inverse Metrics

**Figure 8:**
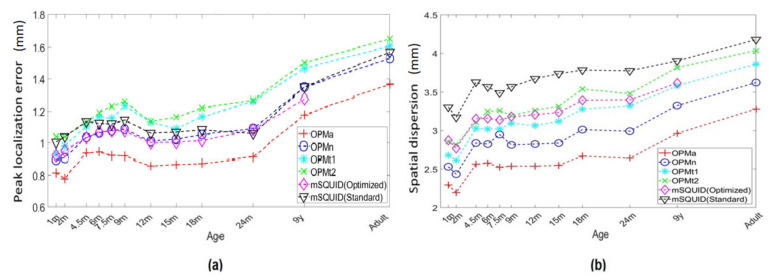
Comparison of array configurations for L2-MNE estimation. (**a**) PLE and (**b**) SD.

Figure 8 shows the resolution metrics: peak localization error (PLE) and spatial deviation (SD) of the L2-MNE estimation for PSFs or cross-talk functions (CTFs). The resolution metrics indicate that the resolution was greater for OPMa than for SQUID. The SD was also lower for the OPMa array; for the adult head model, it was 0.83 lower than with the mSQUID. For OPMn arrays, the SD was 0.95 and 0.93 times lower than those for the OPMt_1_ and OPMt_2_ arrays, respectively, and the PLE for OPMa was 0.87 times lower than that for SQUIDs. For OPMn arrays, the PLE was 0.95 and 0.92 times lower than those for the OPMt_1_ and OPMt_2_ arrays, respectively.

For all the metrics presented above, a paired t-test showed a significant difference between OPMa and mSQUID (optimized) or mSQUID (standard), and between OPMn and mSQUID (optimized) or mSQUID (standard) (*p* < 0.001 in all cases).

We also performed an ANOVA analysis test for the 12 subjects; for each metric (topography power, sensitivity map, DLE and SD), we computed the difference between OPMa and mSQUID (optimized) or mSQUID (standard). The ANOVA showed a significant effect of age, meaning that the OPMa–mSQUID (optimized) or OPMa–mSQUID (standard) difference as a function of subject age was statistically significant (*p* < 0.001). Post hoc comparisons of age groups revealed that differences between OPMa and mSQUID (optimized) or mSQUID (standard) were still statistically significant when comparing the adult with all the younger age groups. This was also true for most of the comparisons between the younger age groups (see the Supplementary Materials for more details). These findings suggested strongly that the gain achieved with OPMa is even greater with smaller heads—even though we considered the optimal situation when the MEG helmet fits the head well and is not solely translated along the z-axis.

## 4. Discussion

The present study had three main components: (i) a simulation of the brain’s magnetic field, as recorded by a ^4^He OPM along the three measurement axes for near-radial and tangential dipoles located within the somesthetic cortex; (ii) a quantitative assessment of the added value of a ^4^He OPM array for recording the brain’s magnetic field; and (iii) a comparison of the added value of a ^4^He OPM in subjects of various ages (from an infant to an adult).

### 4.1. Single Dipole 2D Topographies

In the first part of our study, we simulated the brain’s magnetic field as measured by a ^4^He OPM array for a single dipole case with near-radial and tangential orientations. The ^4^He OPM is notably characterized by its three measurement axes. One can therefore expect this new type of sensor to extract more information from various brain sources, and notably those with a near-radial orientation. It is generally acknowledged that these sources are not well recorded by standard SQUID-based MEG, since the latter only measures the radial brain’s magnetic field (from tangential brain sources). However, a previous study [35], in which SQUID sensors were combined along three axes, demonstrated that near-radial dipolar activity can be reconstructed from three-component MEG measurements. Our present results showed that activity related to the near-radial dipole should be clearly visible on 2D maps for both tangential axes. It is noteworthy that the two tangentially oriented dipoles also resulted in MEG activity recorded on the tangential axes—suggesting that the additional measurement axes provide more information on these dipole orientations. This finding is in line with the literature data on source modeling for the brain and the heart [35,36,37,38,39].

To compensate for MEG’s low sensitivity to radially oriented brain sources, the MEG data are usually combined with much more sensitive EEG measurements [32]. A three-axis measurement of the brain’s magnetic field should compensate (at least in part) for this limitation of today’s MEG devices. Another advantage of a three-axis measurement is better de-noising. This has been investigated by adding additional tangential sensors to a conventional SQUID array [39]. This source space separation method yielded a 100% increase in software shielding capability. Furthermore, recent preliminary results obtained with the first commercial three-axis alkali OPM highlighted a marked improvement in eliminating artifacts caused by head movement [40].

### 4.2. Advantages of the ^4^He OPM Array, and Comparisons with Previous Simulations of Alkali OPMs

Here, we only discuss the results obtained for the adult head model, for easier comparison with previous simulation studies of alkali OPMs and adult head models [14,15]. Although we studied a ^4^He OPM, we nevertheless considered a 102 SQUID magnetometer array in the comparison.

Previously published simulations of OPMs have highlighted the device’s advantages for MEG imaging. The simulations focused on the alkali OPM, the first commercial OPM that was usable for MEG. Our present simulation is the first to have assessed the advantages of using the new ^4^He OPM, despite the latter’s lower sensitivity (40 fT/$\sqrt{\mathrm{Hz}}$ here, vs. 10 fT/$\sqrt{\mathrm{Hz}}$ for alkali OPMs in the literature). However, ^4^He OPMs have major advantages for use in MEG: (i) they have three measurement axes; (ii) continuous self-compensation for external noise ensures reliable brain field measurements; and (iii) they have a broad bandwidth (direct current to 2 kHz) and a large dynamic (>200 nT). Therefore, ^4^He OPMs are better suited for use in MEG as they can record all brain activities, even at very high frequencies (epileptic seizures, high-frequency oscillations, the somesthetic 600 Hz response, etc.) and are less sensitive to noise in the environment. Since ^4^He OPMs operate at room temperature (no heating or cooling required), they can be placed close to the scalp—thus minimizing the distance between the brain sources and the sensor. In this part of the discussion, we will compare the results obtained with the adult head model to those obtained by Livanainen et al. [15], whenever possible.

When considering the metrics for topography power, we found that the OPMa array performed significantly better than the SQUID array. This finding is in line with previous simulations of alkali OPMs [14,15]. Livanainen et al. [15] reported that the average relative power (vs. an mSQUID) was 7.5 for nOPM and 5.3 for tOPM. Our results for a ^4^He OPM showed an 8.9-fold gain for aOPM (relative to an mSQUID) with the adult head model. Likewise, the OPMa outperformed the SQUID array with regard to total information capacity and sensitivity. Regarding OPMn alone, (the most technically similar to an mSQUID array), OPMn also brought benefits compared to SQUID, except for when it came to the total information. This result differs from what has been previously reported for alkali OPM, where both of the OPM arrays (nOPM and tOPM) provided more information than SQUID. This can be explained by the lower sensitivity of ^4^He OPM, set to 40 fT/$\sqrt{\mathrm{Hz}}$ in this simulation study. However, combining all measurement axes, the aOPM array conveyed significantly more information than SQUID. As with the alkali OPM simulations, the tangential OPM (*t*_1_ and *t*_2_ OPM together) provided more information than nOPM did. This can be easily explained by the number of sensors (204 for tOPM vs. 102 for nOPM). However, if only one tangential axis is considered (*t*_1_ or *t*_2_ OPM, see Figure 5), the total information was (as expected) lower than for nOPM. This also means that normal and tangential measurement axes carry independent information. Since the total information conveyed by aOPM was higher than nOPM and tOPM separately, these measurements are not redundant. This finding is in line with previous studies, in which biomagnetic source modeling was more accurate when a three-axis measurement of the brain or cardiac magnetic field was available [35,38,39].

Our sensitivity map results also suggest that three-axis measurement provides a more exhaustive recording of the brain’s magnetic field. The results revealed that the OPMa array was more sensitive to tangentially oriented sources ($\lambda_{min}$), relative to an mSQUID. These sources are poorly visualized by current MEG systems; this is mainly why EEG is usually combined with MEG to improve brain activity recordings. Ahlfors et al. [32] performed a quantitative assessment of the contributions of radial vs. tangential sources to MEG and EEG, by using the sensitivity map metric originally developed by Huang et al. [41]. A gain in sensitivity to radially oriented sources ($\lambda_{max})$, preferentially recorded by mSQUID is also observed with OPMa, mainly for deep sources. This increased sensitivity of three axes OPM array to various sources orientations has never been reported in previous studies on alkali OPM [14,15]. This result suggests that the three-axis ^4^He OPM array simulated here can record brain sources more exhaustively. In principle, combined EEG recording should be less frequently required with this kind of OPM array. However, this hypothesis needs to be confirmed in a comparative study of real brain recordings by ^4^He OPMs combined (or not) with EEG.

In the present work, we assessed the respective contributions of volume and primary currents to the signals measured by OPM and SQUIDs. Our results are in line with those previous simulations of alkali OPMs [15]. The PV values (for the signal measured by tangential axes of ^4^He OPM) close to one (1.02 for *t*_1_ and *t*_2_ OPM) indicated that the volume and primary currents make equivalent contributions. This can be viewed as an advantage as it suggests that tangential axes can measure signals usually recorded by EEG; the latter technique is more sensitive to volume currents, while MEG is more sensitive to primary currents). This finding is also in line with our sensitivity map results and confirms that the OPM’s tangential axes will probably provide better recordings of brain sources poorly visualized by current SQUID MEG and that require the combination of EEG and MEG [32]. The volume current’s contribution to the tangential axis signal can also be viewed as a drawback as it will mask the primary current. However, our simulated TP values (0.28 for *t*_1_ and *t*_2_ OPM) suggest that the volume current did not totally cancel the primary current. Regarding OPMn, the magnetic field recorded along this axis is mainly related to the primary current’s contribution, since the PV value is much greater than one. However, the volume current still makes a contribution. The SQUID array (MEG) and the OPMn array had similar PV and TP values.

We also assessed the accuracy of brain source localization with a ^4^He OPM sensor array by calculating the PLE and SD metrics introduced by Hauk et al. [42]. Our results showed that the PLE for brain source localization was lower for the OPMn array (2.4 mm) than for the mSQUID (2.8 mm). The gain in source localization accuracy was even greater for the OPMa array (2.3 mm). The SD of the brain source localized with OPMn array was also lower (3.5 mm) than that of the mSQUID (4.2 mm); again, this result was even better for the OPMa (3 mm). On the basis of these current simulations and the L2-norm minimum norm estimate used to compute the brain sources, the ^4^He OPM array was more accurate and yielded more focused brain source localizations. Livanainen et al. [15] used the PSF to assess the advantage of an OPM array with regard to source localization accuracy. The researchers reported that the alkali OPM and SQUID arrays gave similar localization accuracies for minimum-norm estimation, however, they also reported that the OPM array gave a greater spatial resolution. Our results go beyond this statement. This disparity might be due to our use of different metrics (PLE and SD) for the PSF and the CTF.

### 4.3. Advantages of the ^4^He OPM with Infant, Child and Adult Subjects

Another salient result of the present study was the first quantitative assessment of the benefits of an OPM array for use with children and infants, relative to SQUID-based MEG recordings. We distinguished between two uses for all child and infant head models: mSQUID (optimized) corresponded to a MEG helmet of the right size for the subject’s size, with a scalp-sensor distance everywhere of two cm; mSQUID (standard) was more representative of real MEG assessments, in which the top of the head is placed close to the MEG helmet. This distinction is particularly important for children and infants, where the head is markedly smaller than the rigid SQUID MEG helmet and thus the scalp-sensor distance is greater. As mentioned in the Introduction, dedicated baby MEG systems are not widely available; here, mSQUID (optimized) corresponds to these devices. In normal MEG systems (as modelled by our mSQUID (standard) configuration), the top of the child’s or infant’s head is placed against the helmet; thus, the scalp-sensor distance is greater than two cm for the other parts of the head.

Considering the topography power, the total information capacity and the sensitivity metrics, OPMa still outperformed SQUID arrays for all subject ages. As expected, the benefit for the nine-year-old child (relative to the adult head model) was greater for OPMa vs. mSQUID (standard) then for OPMa vs. mSQUID (optimized). The benefit for topography power and sensitivity with the OPMa array vs. mSQUID (standard) was significantly greater for the (smaller) infant head than for the nine-year-old child model. Considering OPMn alone, it is also noteworthy that total information was increased relative to mSQUID standard array for infants, which was not observed for the adult head. These findings are the first quantitative indications of how the OPM array–scalp distance can affect the information content and the sensitivity of MEG recordings in children and infants. It emphasizes the particular value of OPM sensors for MEG recordings in children and infants due to placement on the scalp. A ^4^He OPM operates at room temperature (no heating occurs, in contrast to alkali OPMs) and does not have heat dissipation issues; hence, the device can be placed close to the scalp and so is particularly well suited to MEG recordings in infants and children.

With regard to TP and PV, the results for the OPM tangential axes were similar for adults, children, and infants. However, OPMn gave significantly higher PV values than either mSQUID (standard) or mSQUID (optimized)—showing that the primary current contributes more to MEG signals recorded with OPMn than those recorded with an mSQUID. This result suggests that the primary current contributes more to MEG signals, in view of the higher-amplitude signals typically recorded in children and infants [43,44]. The significant difference between OPMn and mSQUID might be due to the lower sensor-scalp distance, which optimizes the recording of the brain’s magnetic field, notably in infants. Regarding the TP parameter, the values for OPMn were close to one; hence, the volume currents (which are even greater in children and infants as the skull is less ossified) did not mask the greater primary current contribution to the MEG signals (as shown by higher PV values). As expected, these differences were even greater when considering mSQUID (standard) instead of mSQUID (optimized).

The OPMn array, and notably the OPMa array, gave smaller PLE and SD values, thus confirming that OPM arrays provide more accurate, focused brain source localization in children and infants. We observed a significant effect of age on difference between OPMa and mSQUID (optimized) or mSQUID (standard); the smaller the head size, the greater the gain in source location accuracy. Our values showed that the differences in PLE and SD between OPMa and mSQUID (standard) were, respectively, 0.17 mm and 0.9 mm for the nine-year-old child, while these differences were 0.18 mm and 1 mm for the one-month-old baby, corresponding to an increase in source localization accuracy of 9.15% and focality of 6.71%.

## 5. Conclusions and Perspectives

Overall, our results confirm the significant advantages of a three-axis 102-sensor ^4^He OPM array, relative to an mSQUID counterpart. We extended our simulations to children and infants of different ages and found that the added value achieved with an OPM array is even greater at smaller head sizes; indeed, the brain source–sensor distance becomes increasingly critical. These results were obtained by simulating a ^4^He OPM with a sensitivity of 40 fT/$\sqrt{\mathrm{Hz}}$. Despite the ^4^He OPM array’s low sensitivity, its three-axis measurement and its proximity to the scalp meant that it outperformed the mSQUID. Our results were also in line with previous simulations of alkali OPM sensors. Thanks to ongoing development, the ^4^He OPM’s low sensitivity is now rising; a value of 30 fT/$\sqrt{\mathrm{Hz}}$ was reported recently [22]. This increase in sensitivity might lead to a further improvement, relative to the results reported here.

The present simulation study compared a 102-sensor OPM array with the 102-sensor mSQUID array found in commercial MEG devices (MEGIN, Espoo, Finland) and previously published simulations of alkali OPM arrays. Our work should now be extended by simulating data with smaller three-axis ^4^He OPM arrays, in order to define the minimum number of OPMs required to achieve much the same performance levels as a standard mSQUID array. Furthermore, other MEG devices (such as those produced by CTF, San Diego, CA) feature a larger number of mSQUIDs. Lastly, it would be interesting to extend this work on the ^4^He OPM array to fetal brain data. However, modelling the womb and developing a fetal head model would be particularly challenging.

## Figures and Tables

**Table 1:** Sensor models used in the present study. N is the number of integration points, w is the weight, σ is the noise level, and d is the sensor-scalp distance.

Sensor Type	N	w	σ	d
**OPM**	4	$\frac{1}{4}$	$40\;\mathrm{fT}\sqrt{\mathrm{Hz}}$	3 mm
**SQUID magnetometer**	4	4	$5\;\mathrm{fT}\sqrt{\mathrm{Hz}}$	20 mm

## Data Availability

The data that support the findings of this study are available from the corresponding author, upon reasonable request.

## Supplementary Materials

The following supporting information can be downloaded at: https://www.mdpi.com/article/10.3390/s22083093/s1, Supplementary_material.pdf.

## References

[1] Kakigi R., Koyama S., Hoshiyama M., Kitamura Y., Shimojo M., Watanabe S. (1995). Pain-related magnetic fields following painful CO_2_ laser stimulation in man. Neurosci. Lett..

[2] Darvas F., Pantazis D., Kucukaltun-Yildirim E., Leahy R. (2004). Mapping human brain function with MEG and EEG: Methods and validation. NeuroImage.

[3] Cheyne D., Bostan A.C., Gaetz W., Pang E.W. (2007). Event-related beamforming: A robust method for presurgical functional mapping using MEG. Clin. Neurophysiol..

[4] Colon A.J., Ossenblok P., Nieuwenhuis L., Stam K.J., Boon P. (2009). Use of Routine MEG in the Primary Diagnostic Process of Epilepsy. J. Clin. Neurophysiol..

[5] Hill R.M., Boto E., Holmes N., Hartley C., Seedat Z.A., Leggett J., Roberts G., Shah V., Tierney T.M., Woolrich M.W. (2019). A tool for functional brain imaging with lifespan compliance. Nat. Commun..

[6] Okada Y., Hämäläinen M., Pratt K., Mascarenas A., Miller P., Han M., Paulson D. (2016). BabyMEG: A whole-head pediatric magnetoencephalography system for human brain development research. Rev. Sci. Instrum..

[7] Cooper R., Winter A., Crow H., Walter W. (1965). Comparison of subcortical, cortical and scalp activity using chronically indwelling electrodes in man. Electroencephalogr. Clin. Neurophysiol..

[8] DeLucchi M., Garoutte B., Aird R.B. (1962). The scalp as an electroencephalographic averager. Electroencephalogr. Clin. Neurophysiol..

[9] Baillet S. (2017). Magnetoencephalography for brain electrophysiology and imaging. Nat. Neurosci..

[10] Budker D., Romalis M. (2007). Optical magnetometry. Nat. Phys..

[11] Allred J.C., Lyman R.N., Kornack T.W., Romalis M.V. (2002). High-sensitivity atomic magnetometer unaffected by spin-exchange relaxation. Phys. Rev. Lett..

[12] Mhaskar R., Knappe S., Kitching J. (2012). A low-power, high-sensitivity micromachined optical magnetometer. Appl. Phys. Lett..

[13] Shah V.K., Wakai R.T. (2013). A compact, high performance atomic magnetometer for biomedical applications. Phys. Med. Biol..

[14] Boto E., Bowtell R.W., Kruger P., Fromhold T.M., Morris P.G., Meyer S.S., Barnes G.R., Brookes M.J. (2016). On the potential of a new generation of magnetometers for MEG: A beamformer simulation study. PLoS ONE.

[15] Iivanainen J., Stenroos M., Parkkonen L. (2017). Measuring MEG closer to the brain: Performance of on-scalp sensor arrays. NeuroImage.

[16] Hill R.M., Boto E., Rea M., Holmes N., Leggett J., Coles L.A., Papastavrou M., Everton S.K., Hunt B.A., Sims D. (2020). Multi-channel whole-head OPM-MEG: Helmet design and a comparison with a conventional system. NeuroImage.

[17] Holmes N., Leggett J., Boto E., Roberts G., Hill R.M., Tierney T.M., Shah V., Barnes G.R., Brookes M.J., Bowtell R. (2018). A bi-planar coil system for nulling background magnetic fields in scalp mounted magnetoencephalography. NeuroImage.

[18] Seymour R.A., Alexander N., Mellor S., O’Neill G.C., Tierney T.M., Barnes G.R., Maguire E.A. (2021). Interference suppression techniques for OPM-based MEG: Opportunities and challenges. arXiv.

[19] Beato F., Belorizky E., Labyt E., Le Prado M., Palacios-Laloy A. (2018). Theory of a He4 parametric-resonance magnetometer based on atomic alignment. Phys. Rev. A.

[20] Labyt E., Corsi M.-C., Fourcault W., Palacios Laloy A., Bertrand F., Lenouvel F., Cauffet G., Le Prado M., Berger F., Morales S. (2019). Magnetoencephalography with Optically Pumped 4He Magnetometers at Ambient Temperature. IEEE Trans. Med. Imaging.

[21] Fourcault W., Romain R., Le Gal G., Bertrand F., Josselin V., Le Prado M., Labyt E., Palacios-Laloy A. (2021). Helium-4 magnetometers for room-temperature biomedical imaging: Toward collective operation and photon-noise limited sensitivity. Opt. Express.

[22] Romain R., Mitryukovskiy S., Fourcault W.V., Josselin M.L., Prado E., Labyt A., Palacios L. A metastable helium-4 OPM for medical imaging. Proceedings of the 9th Workshop on Optically Pumped Magnetometers (WOPM-2021).

[23] Dale A.M., Fischl B., Sereno M.I. (1999). Cortical surface-based analysis. I. Segmentation and surface reconstruction. NeuroImage.

[24] Gramfort A., Papadopoulo T., Olivi E., Clerc M. (2010). OpenMEEG: Opensource software for quasistatic bioelectromagnetics. Biomed. Eng. Online.

[25] Geddes L.A., Baker L.E. (1967). The specific resistance of biological material—A compendium of data for the biomedical engineer and physiologist. Med. Biol. Eng. Comput..

[26] Tadel F., Baillet S., Mosher J.C., Pantazis D., Leahy R.M. (2011). Brainstorm: A user-friendly application for MEG/EEG analysis. Comput. Intell. Neurosci..

[27] Hämäläinen M.S., Ilmoniemi R. (1994). Interpreting magnetic fields of the brain: Minimum norm estimates. Med. Biol. Eng. Comput..

[28] Dale A.M., Sereno M.I. (1993). Improved localizadon of cortical activity by combining EEG and MEG with MRI cortical surface reconstruction: A linear approach. J. Cogn. Neurosci..

[29] Kemppainen P.K., Ilmoniemi R.J. (1989). Channel capacity of multichannel magnetometers. Advances in Biomagnetism.

[30] Nenonen M., Kajola J., Simola J., Ahonen A. Total information of multichannel MEG sensor arrays. Proceedings of the 14th international conference on biomagnetism (Biomag2004).

[31] Ritchie D. (1986). Shannon and Weaver: Unravelling the paradox of information. Commun. Res..

[32] Ahlfors S.P., Han J., Belliveau J.W., Hämäläinen M.S. (2010). Sensitivity of MEG and EEG to Source Orientation. Brain Topogr..

[33] Yao J., Dewald J.P. (2005). Evaluation of different cortical source localization methods using simulated and experimental EEG data. NeuroImage.

[34] Molins A., Stufflebeam S., Brown E., Hämäläinen M. (2008). Quantification of the benefit from integrating MEG and EEG data in minimum ℓ2-norm estimation. NeuroImage.

[35] Haueisen J., Fleissig K., Strohmeier D., Elsarnagawy T., Huonker R., Liehr M., Witte O. (2012). Reconstruction of quasi-radial dipolar activity using three-component magnetic field measurements. Clin. Neurophysiol..

[36] Diekmann V., Becker W., Grözinger B., Jürgens R., Kornhuber C. (1991). A comparison of normal and tangential magnetic field component measurements in biomagnetic investigations. Clin. Phys. Physiol. Meas..

[37] Hochwald B., Nehorai A. (1997). Magnetoencephalography with diversely oriented and multicomponent sensors. IEEE Trans. Biomed. Eng..

[38] Arturi C., Di Rienzo L., Haueisen J. (2004). Information content in single-component versus three-component cardiomagnetic fields. IEEE Trans. Magn..

[39] Nurminen J., Taulu S., Nenonen J., Helle L., Simola J., Ahonen A. (2013). Improving MEG performance with additional tangential sensors. IEEE Trans. Biomed. Eng..

[40] Brookes M.J., Boto E., Rea M., Shah V., Osborne J., Holmes N., Hill R.M., Leggett J., Rhodes N., Bowtell R. (2021). Theoretical advantages of a triaxial optically pumped magnetometer magnetoencephalography system. NeuroImage.

[41] Huang M.-X., Song T., Hagler D.J., Podgorny I., Jousmäki V., Cui L., Gaa K., Harrington D.L., Dale A.M., Lee R.R. (2007). A novel integrated MEG and EEG analysis method for dipolar sources. NeuroImage.

[42] Hauk O., Stenroos M., Treder M. (2019). Towards an Objective Evaluation of EEG/MEG Source Estimation Methods: The Linear Tool Kit. bioRxiv.

[43] Wallois F., Routier L., Heberlé C., Mahmoudzadeh M., Bourel-Ponchel E., Moghimi S. (2021). Back to basics: The neuronal substrates and mechanisms that underlie the electroencephalogram in premature neonates. Neurophysiol. Clin. Neurophysiol..

[44] Mahmoudzadeh M., Dehaene-Lambertz G., Kongolo G., Fournier M., Goudjil S., Wallois F. (2018). Consequence of intraventricular hemorrhage on neurovascular coupling evoked by speech syllables in preterm neonates. Dev. Cogn. Neurosci..

