# Supplementary material for: Performance Analysis of Optically Pumped 4He Magnetometers vs. Conventional SQUIDs: From Adult to Infant Head Models
Source: Sensors (Basel). 2022 Apr 18;22(8):3093. doi: 10.3390/s22083093 (PMC9024855; doi:10.3390/s22083093)

Supplementary material

Topography power

Topography power - Adult

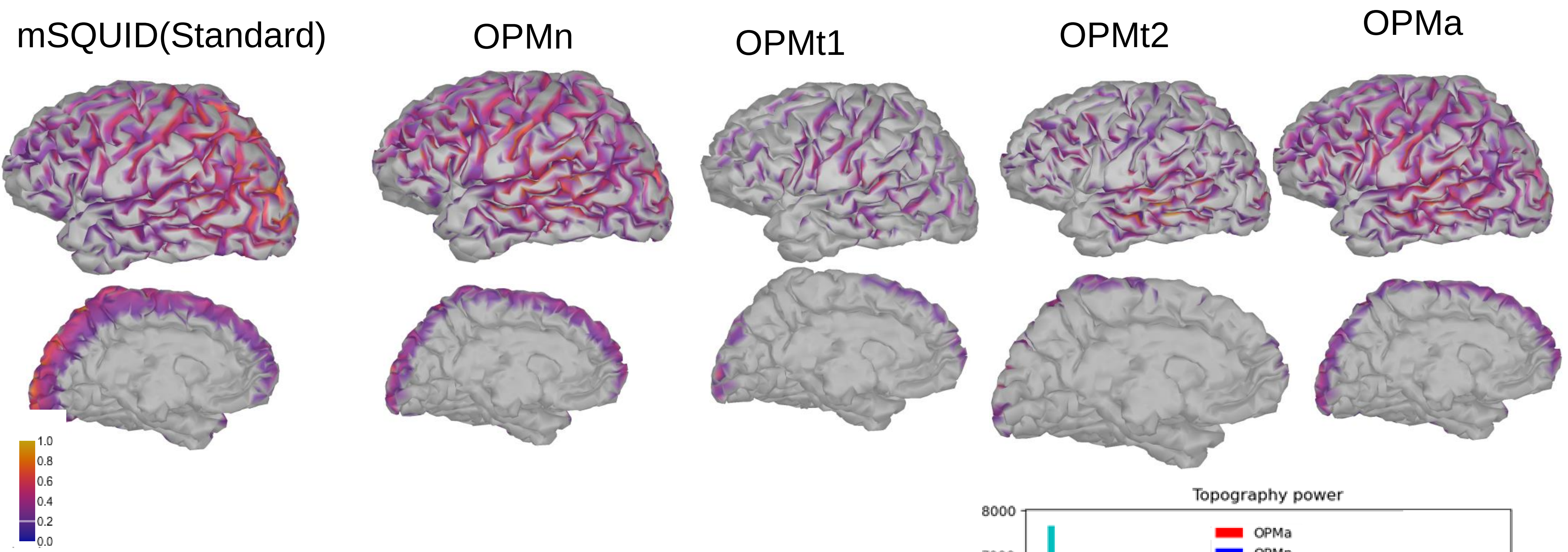

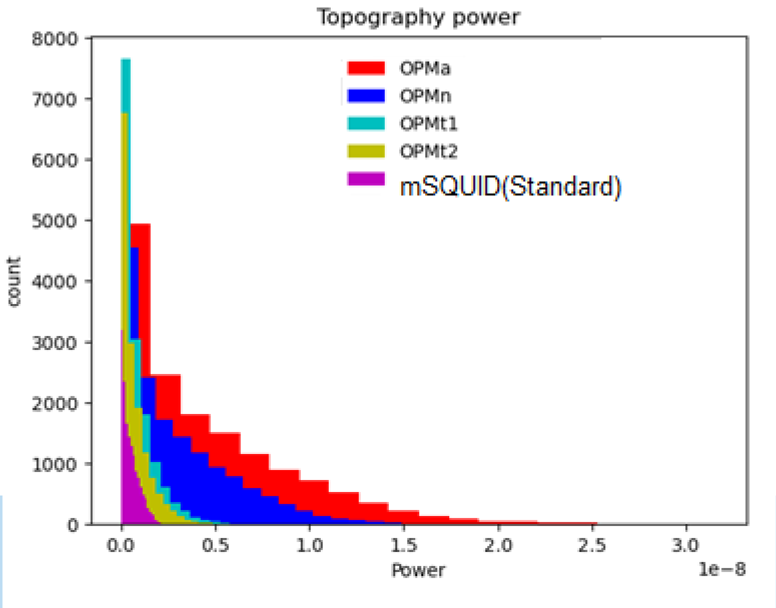

Topography power - 1years

MEG MEGz OPMn OPMt1 OPMt2 OPMa

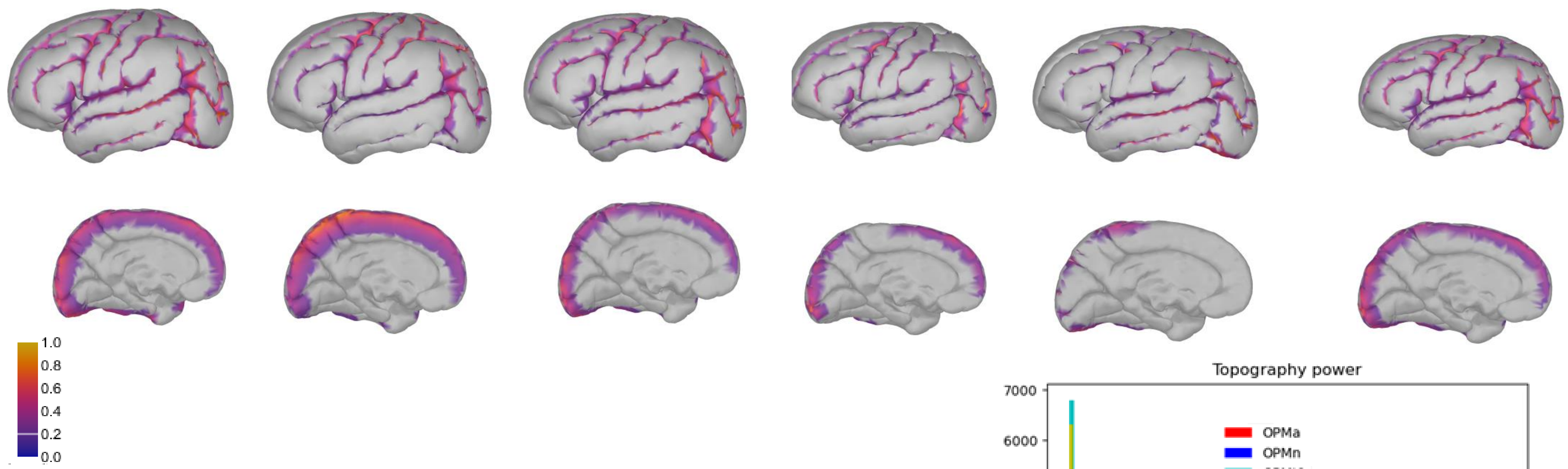

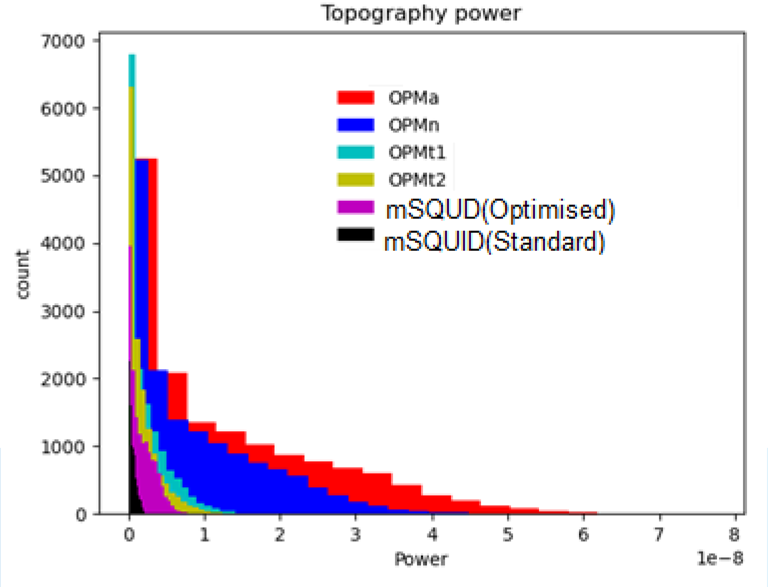

Sensitivity map

Distribution of the sensitivity to three orthogonal source components - Adult

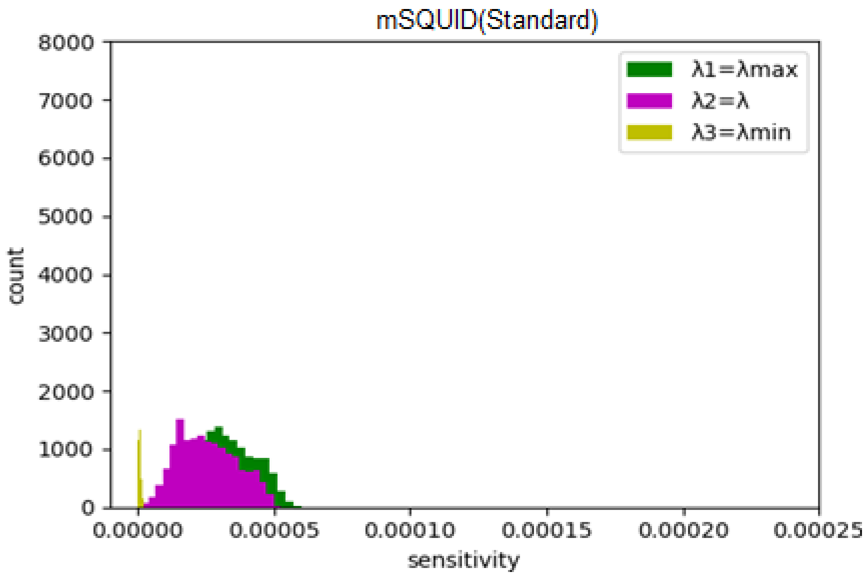

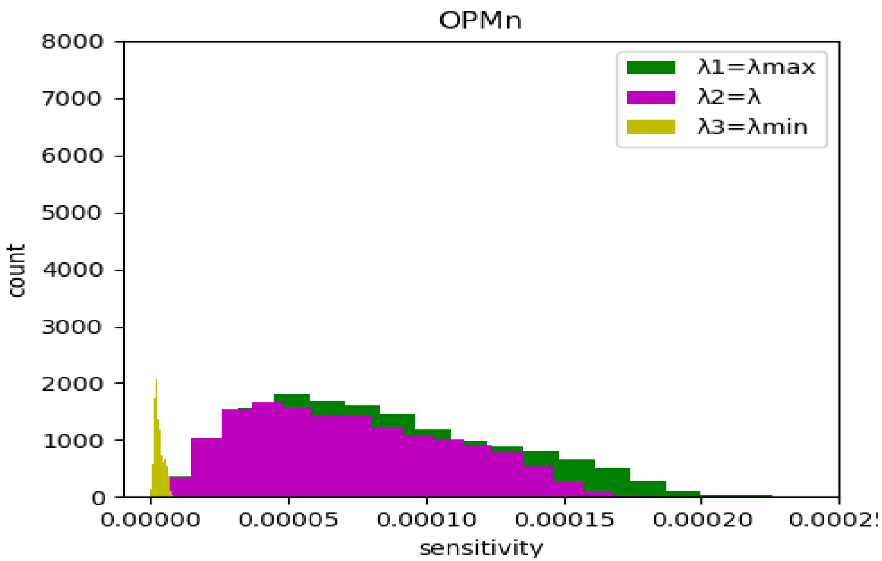

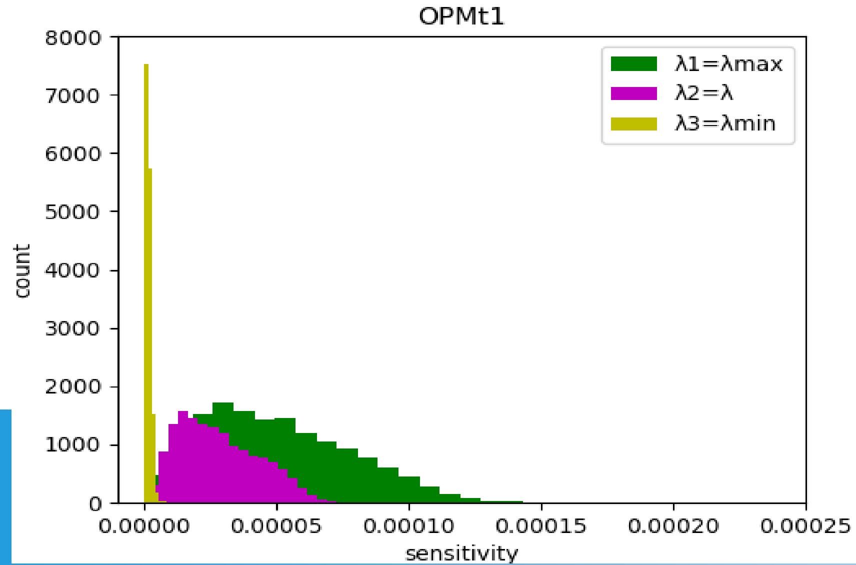

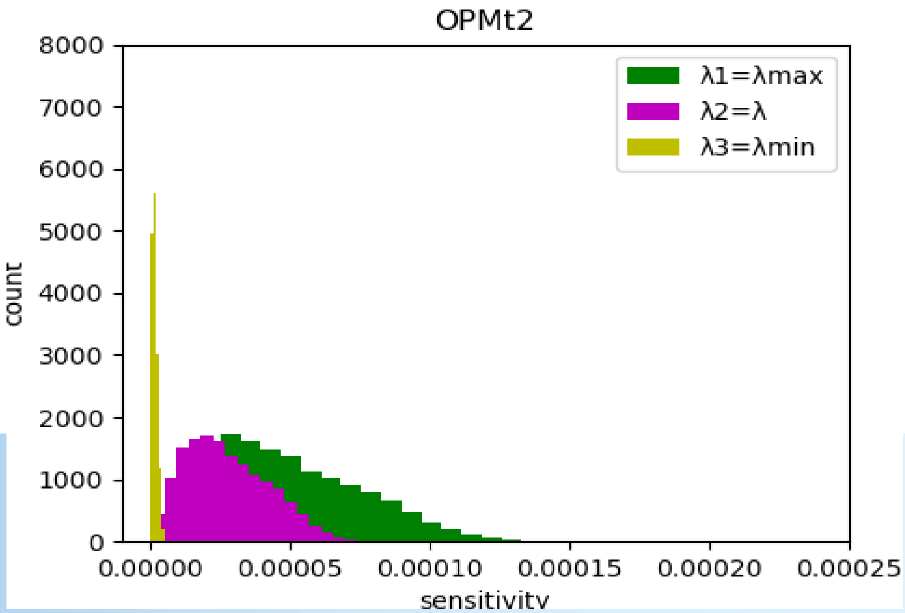

Sensors model – 24 months

mSQUID(Standard)

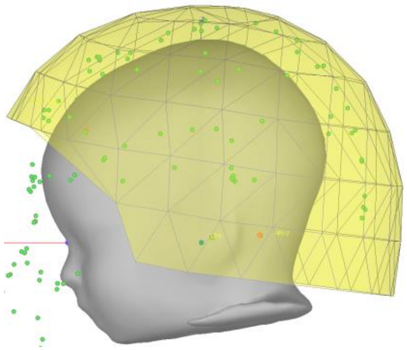

mSQUID(Optimized)

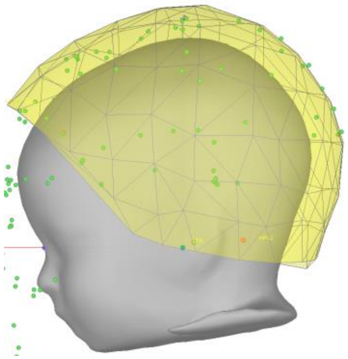

OPM

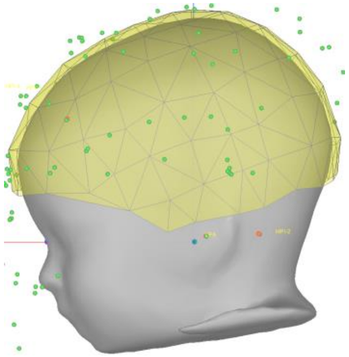

Distribution of the sensitivity to three orthogonal source components – 24 months

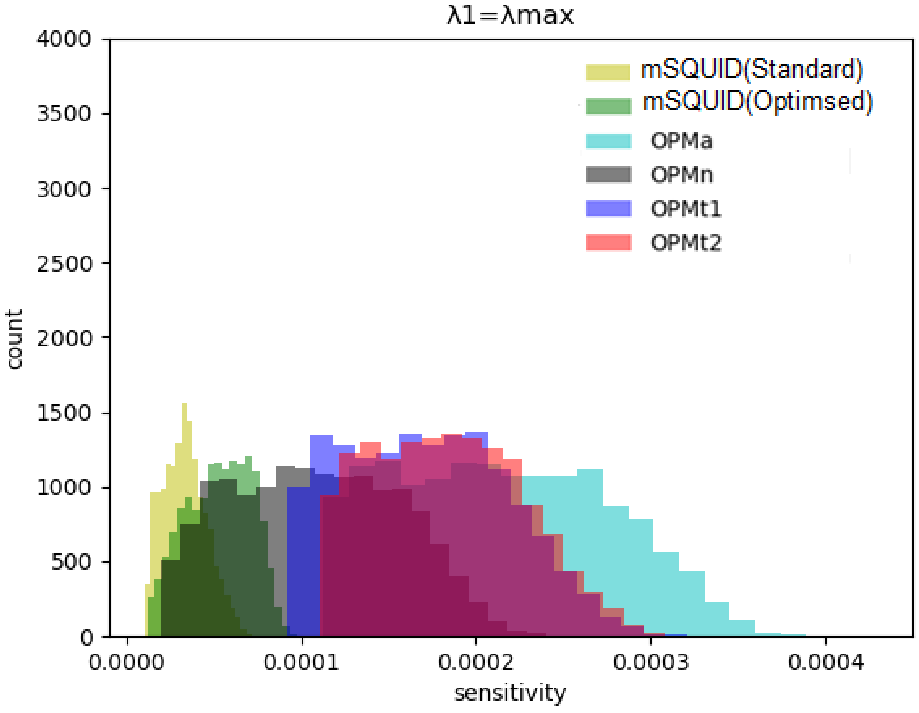

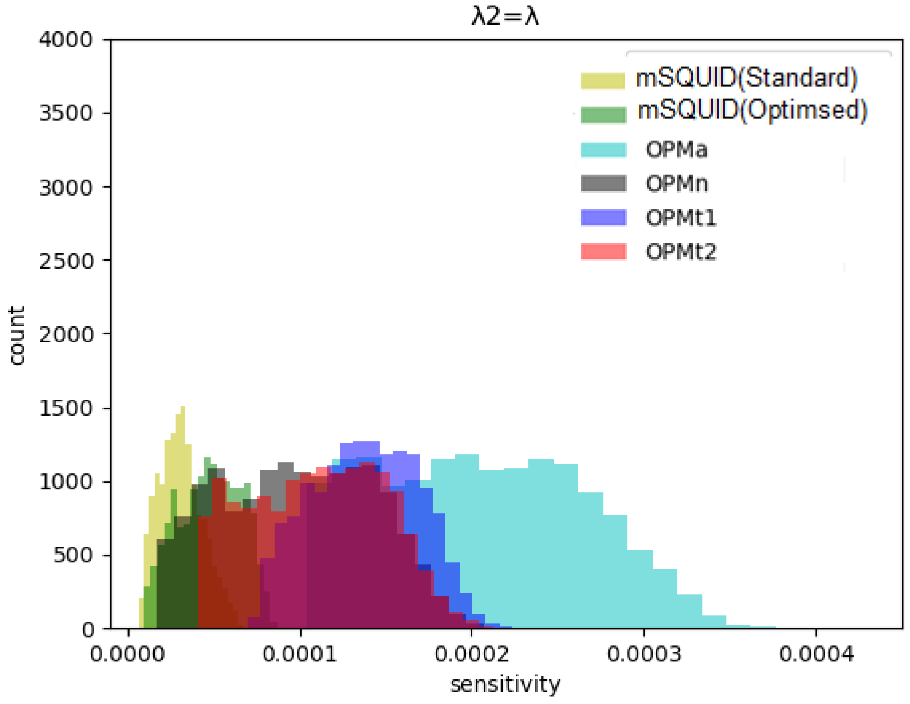

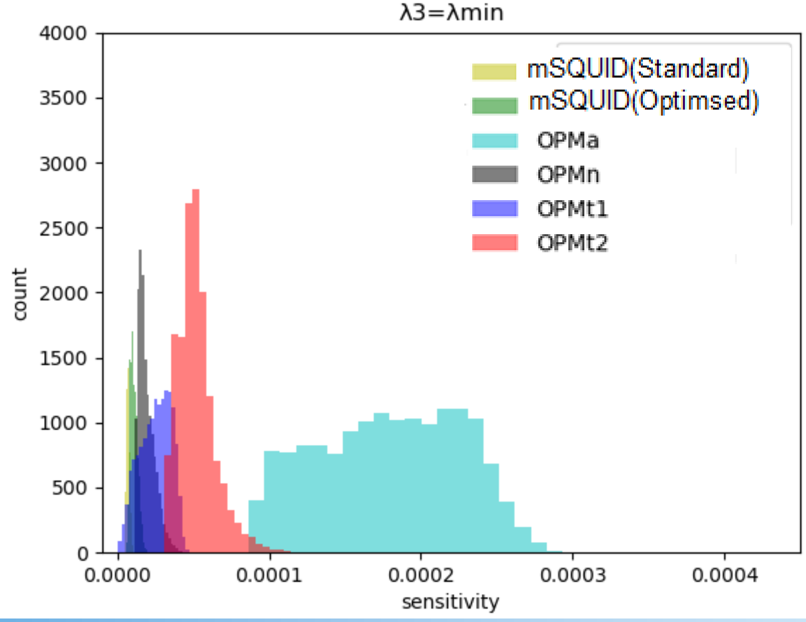

Sensors model – 18 months

mSQUID(Standard)

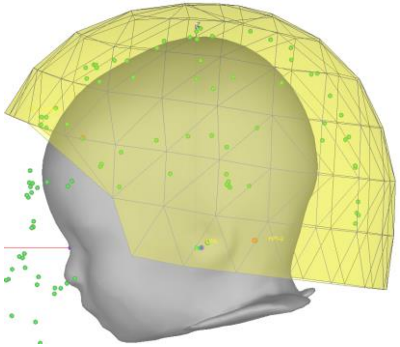

mSQUID(Optimized)

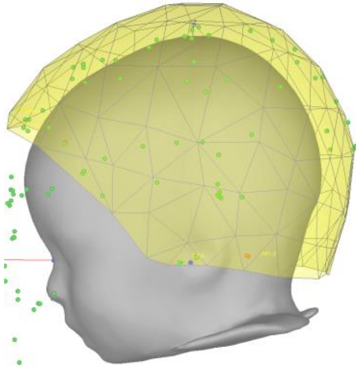

OPM

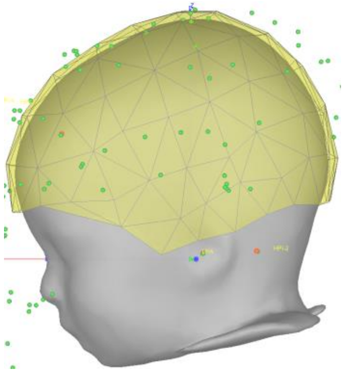

Distribution of the sensitivity to three orthogonal source components – 18 months

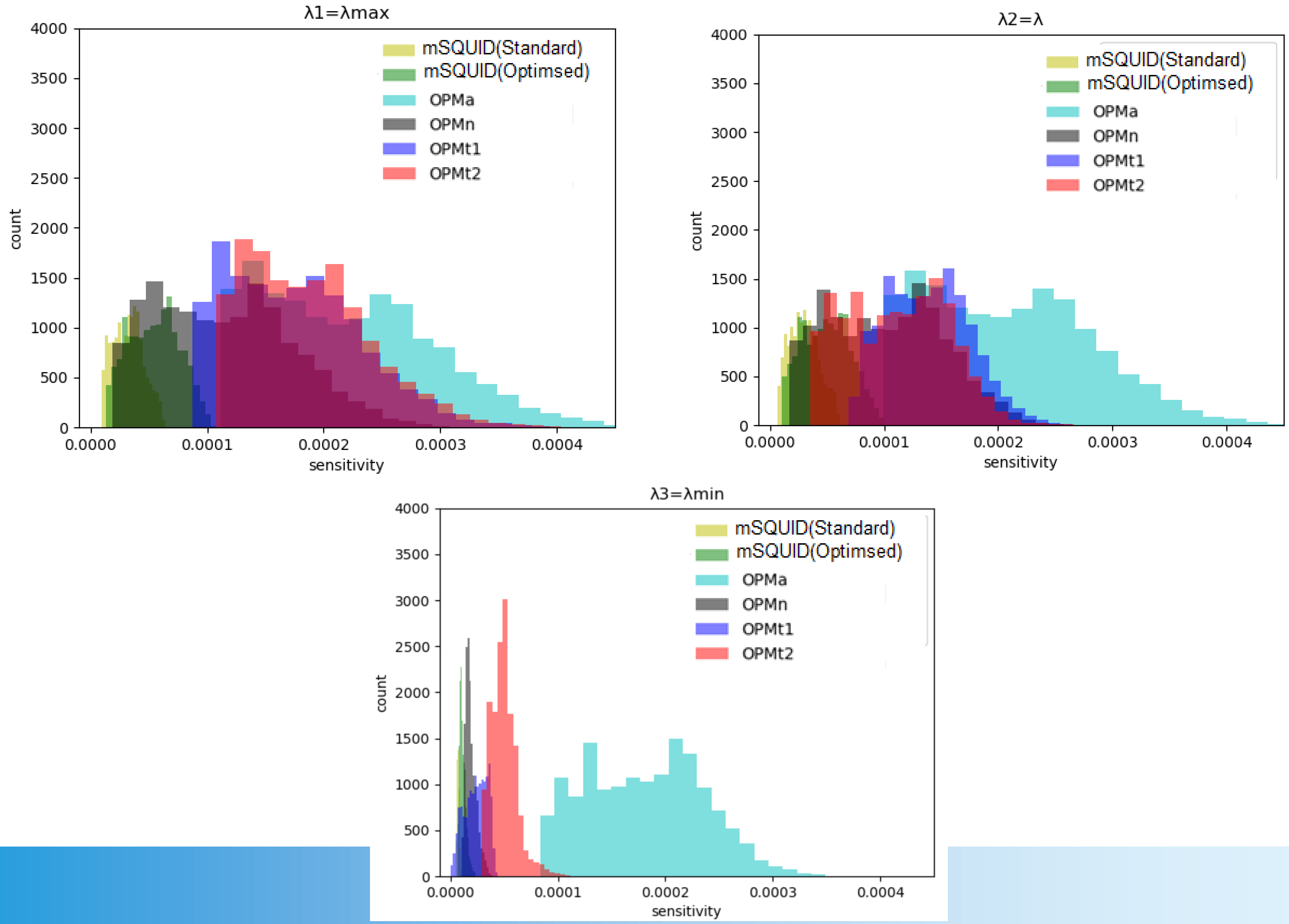

Sensors model – 0.5 months

<u>0,5 months</u>

mSQUID(Standard)

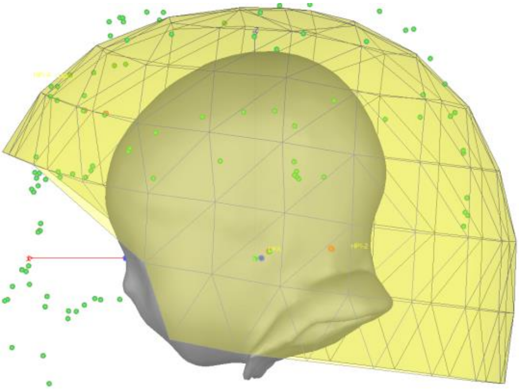

mSQUID(Optimized)

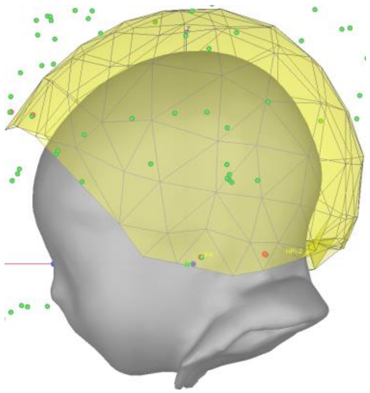

OPM

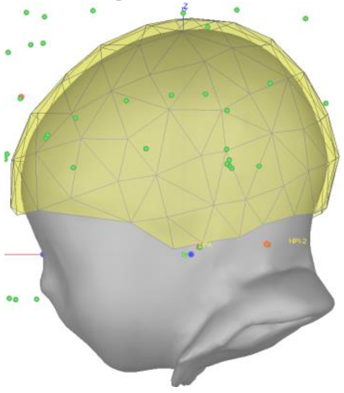

Distribution of the sensitivity to three orthogonal source components – 0.5 months

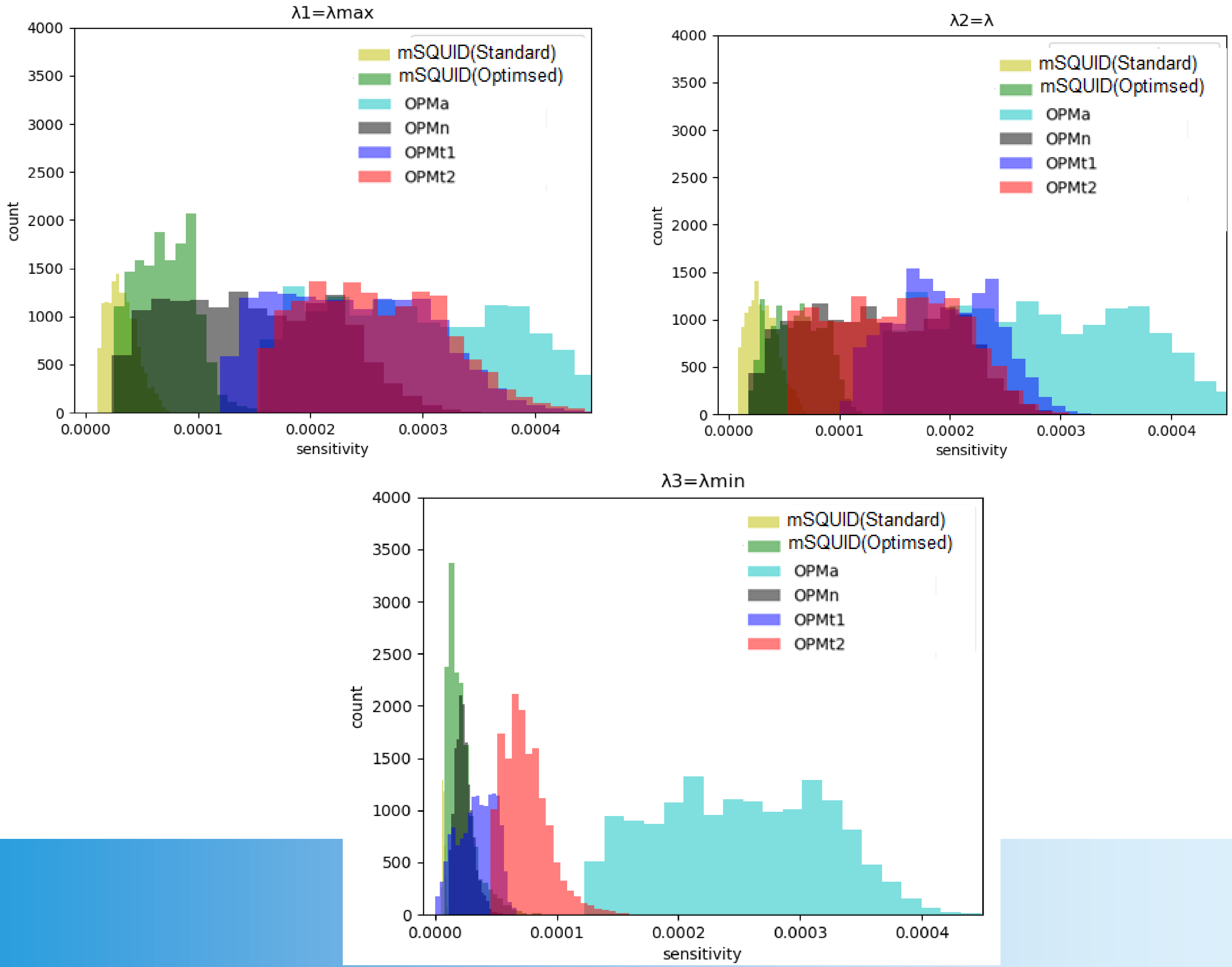

Analysis of Variance (ANOVA)

Topography power (OPMa-mSQUID(Optimized))

ANOVA Table

Source	SS	df	MS	F	Prob>F
Columns	1.07613e-12	12	8.96779e-14	654.22	0
Error	2.67315e-11	195013	1.37075e-16		
Total	2.78076e-11	195025			

Multi compare

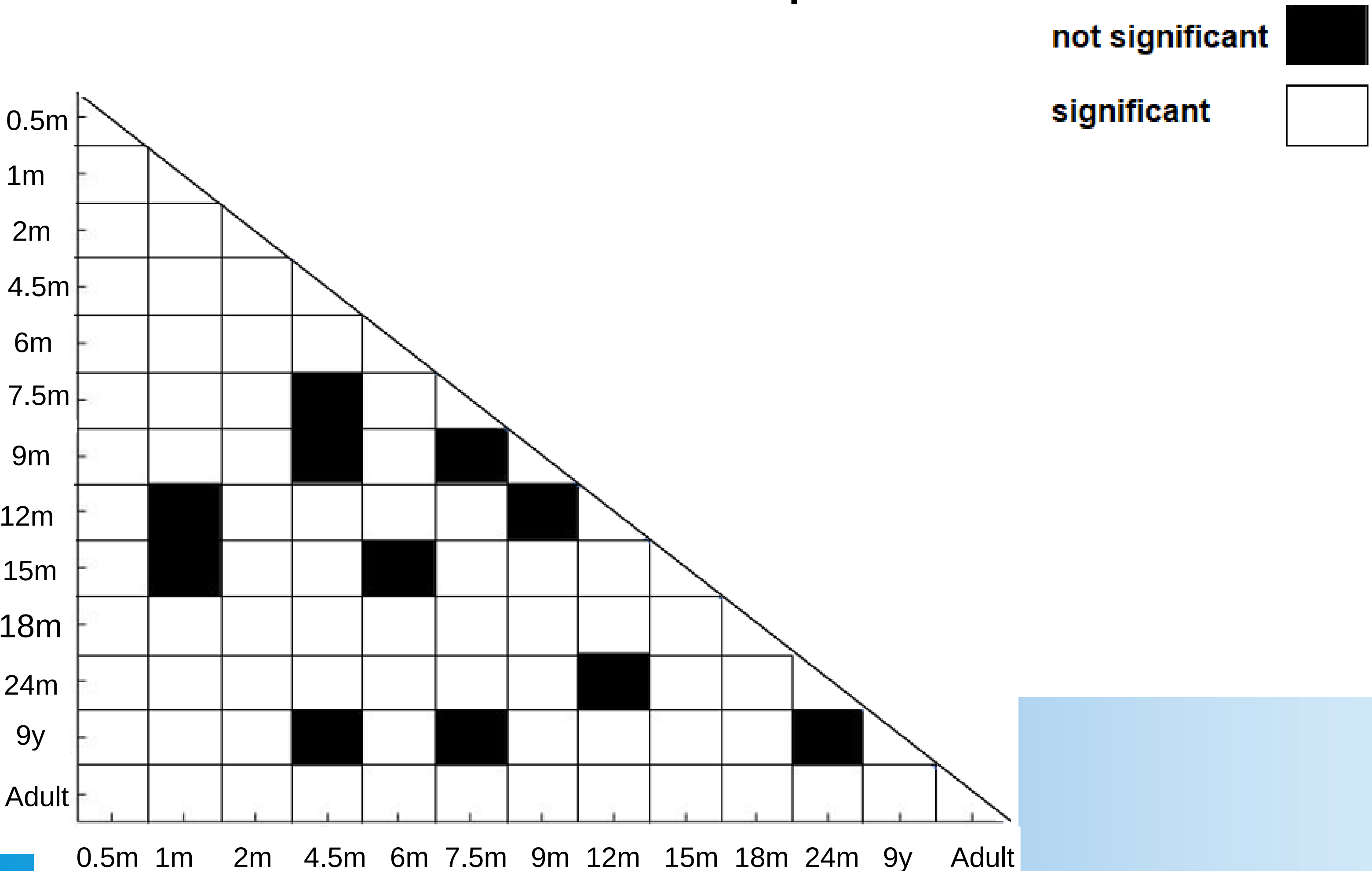

Topography power (OPMa-mSQUID(Standard))

ANOVA Table

Source	SS	df	MS	F	Prob>F
Columns	1.48412e-12	12	1.23677e-13	749.35	0
Error	3.21859e-11	195013	1.65045e-16		
Total	3.36701e-11	195025			

Multi compare

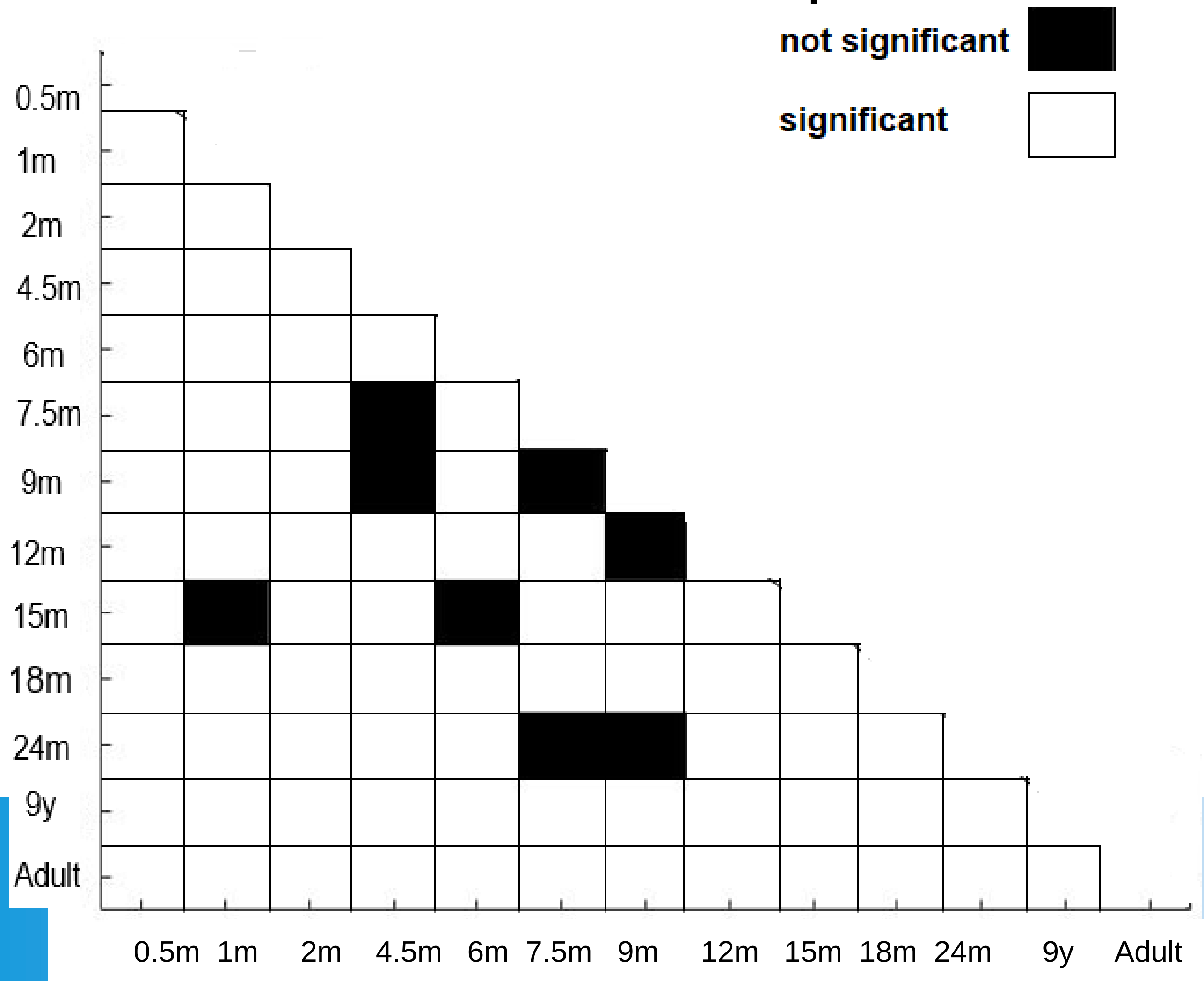

Sensitivity map (OPMa-mSQUID (Optimized))
Lambda_max

ANOVA Table

Source	SS	df	MS	F	Prob>F
Columns	0.00003	12	2.71905e-06	1008.87	0
Error	0.00053	195013	2.69515e-09		
Total	0.00056	195025			

Multi compare

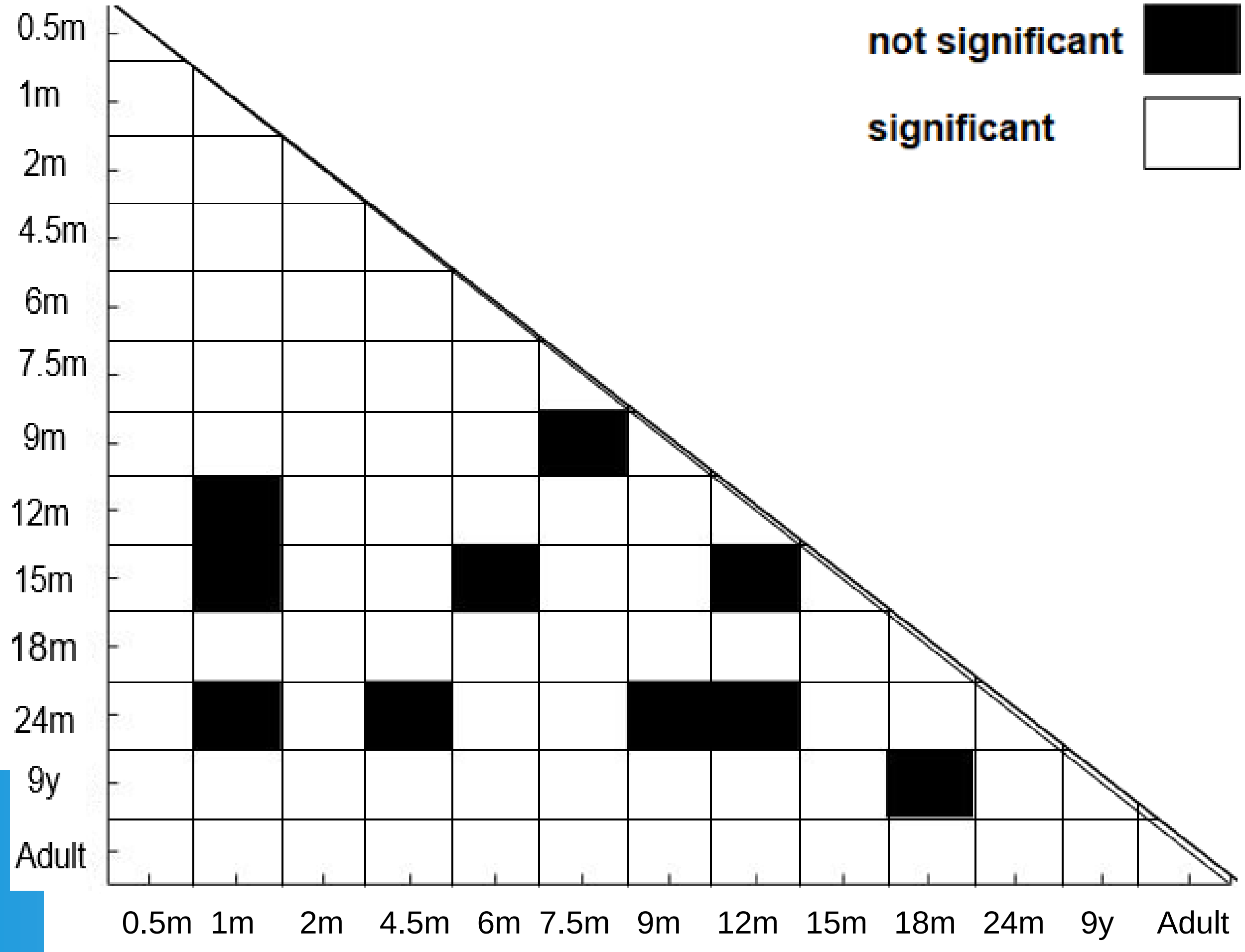

Sensitivity map (OPMa-mSQUID (Optimized)) Lambda_min

ANOVA Table

Source	SS	df	MS	F	Prob>F
Columns	2.24672e-07	12	1.87227e-08	2138.69	0
Error	1.70719e-06	195013	8.75426e-12		
Total	1.93187e-06	195025			

Multi compare

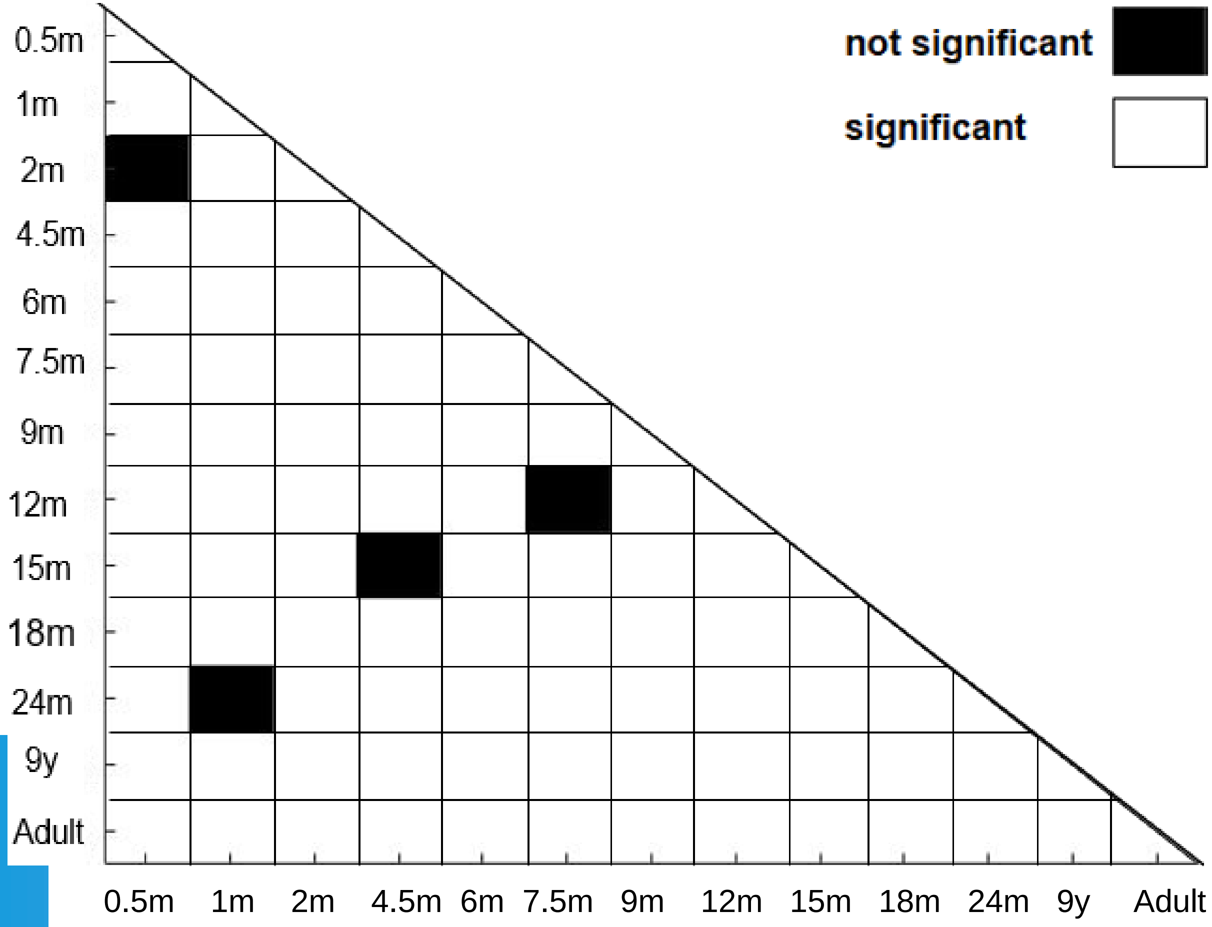

Sensitivity map (OPMa-mSQUID (Standard))
Lambda_max

ANOVA Table

Source	SS	df	MS	F	Prob>F
Columns	0.0001	12	8.15775e-06	2003.32	0
Error	0.00079	195013	4.07211e-09		
Total	0.00089	195025			

Multi compare

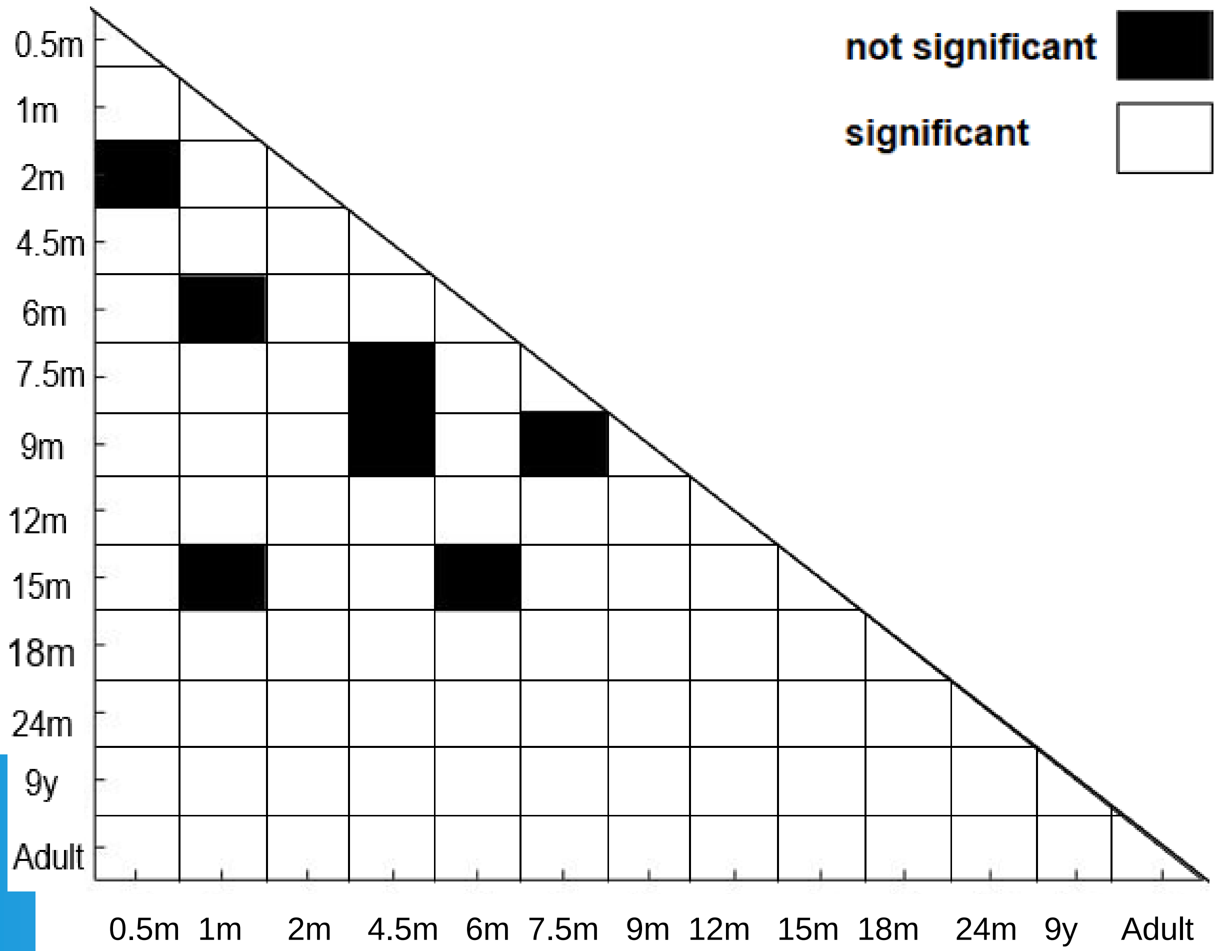

Sensitivity map (OPMa-mSQUID (Standard))
Lambda_min

ANOVA Table

Source	SS	df	MS	F	Prob>F
Columns	4.29405e-07	12	3.57838e-08	3196.61	0
Error	2.18303e-06	195013	1.11943e-11		
Total	2.61244e-06	195025			

Multi compare

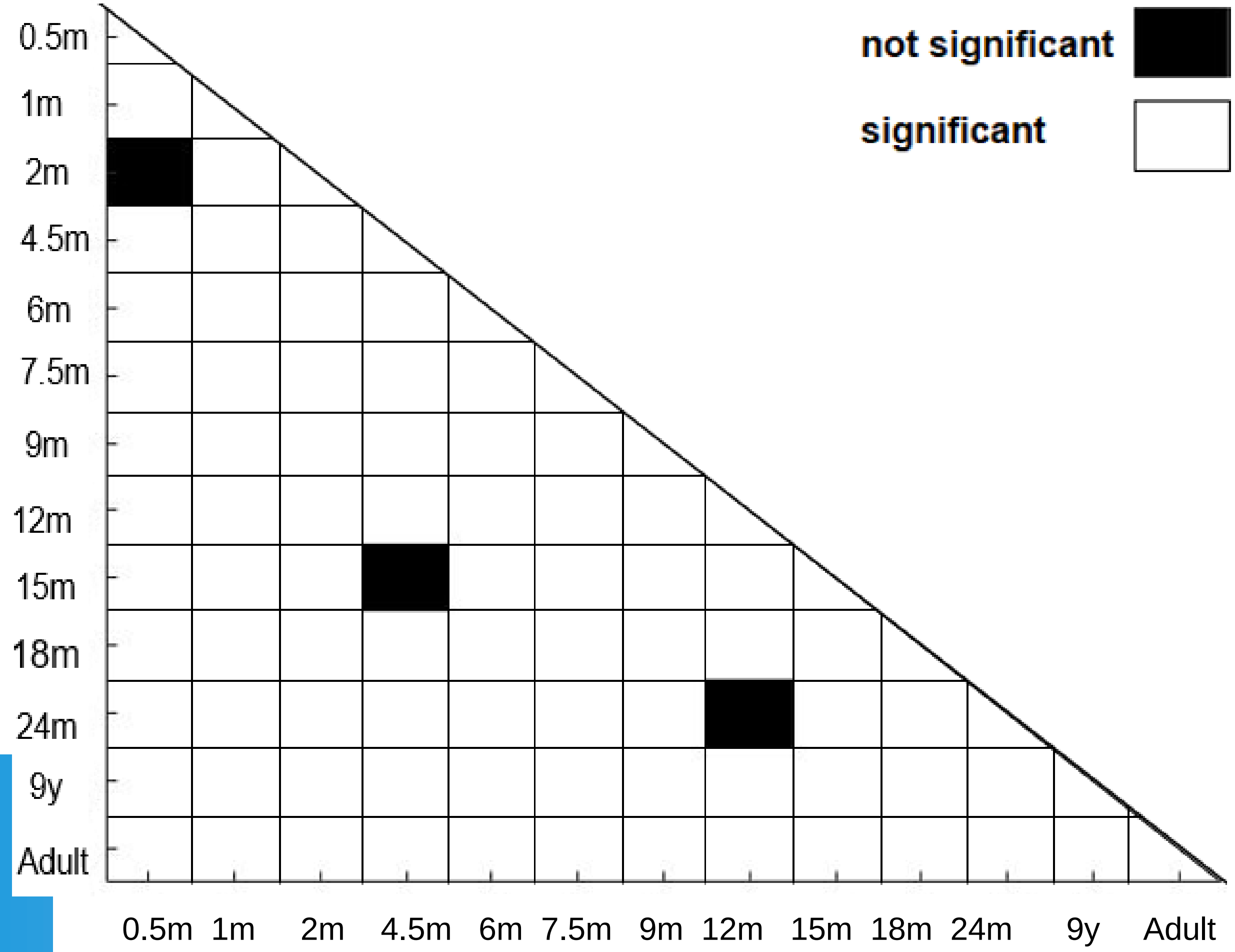

PV (OPMa-mSQUID(Optimized))

ANOVA Table

Source	SS	df	MS	F	Prob>F
Columns	9540.8	12	795.07	399.56	0
Error	388048.4	195013	1.99		
Total	397589.2	195025			

Multi compare

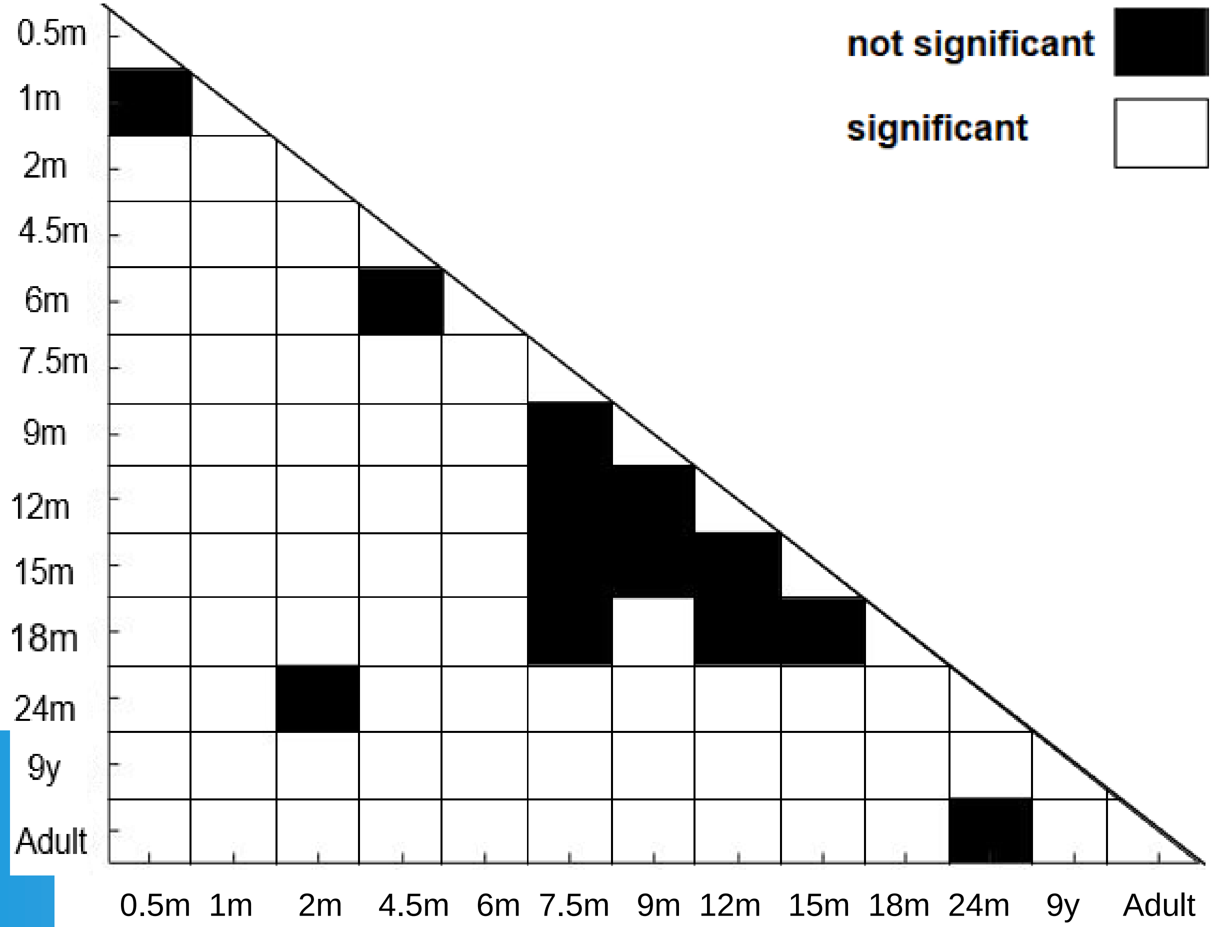

PV (OPMa-mSQUID (Standard))

ANOVA Table

Source	SS	df	MS	F	Prob>F
Columns	12345	12	1028.75	2104.76	0
Error	95317.4	195013	0.49		
Total	107662.4	195025			

Multi compare

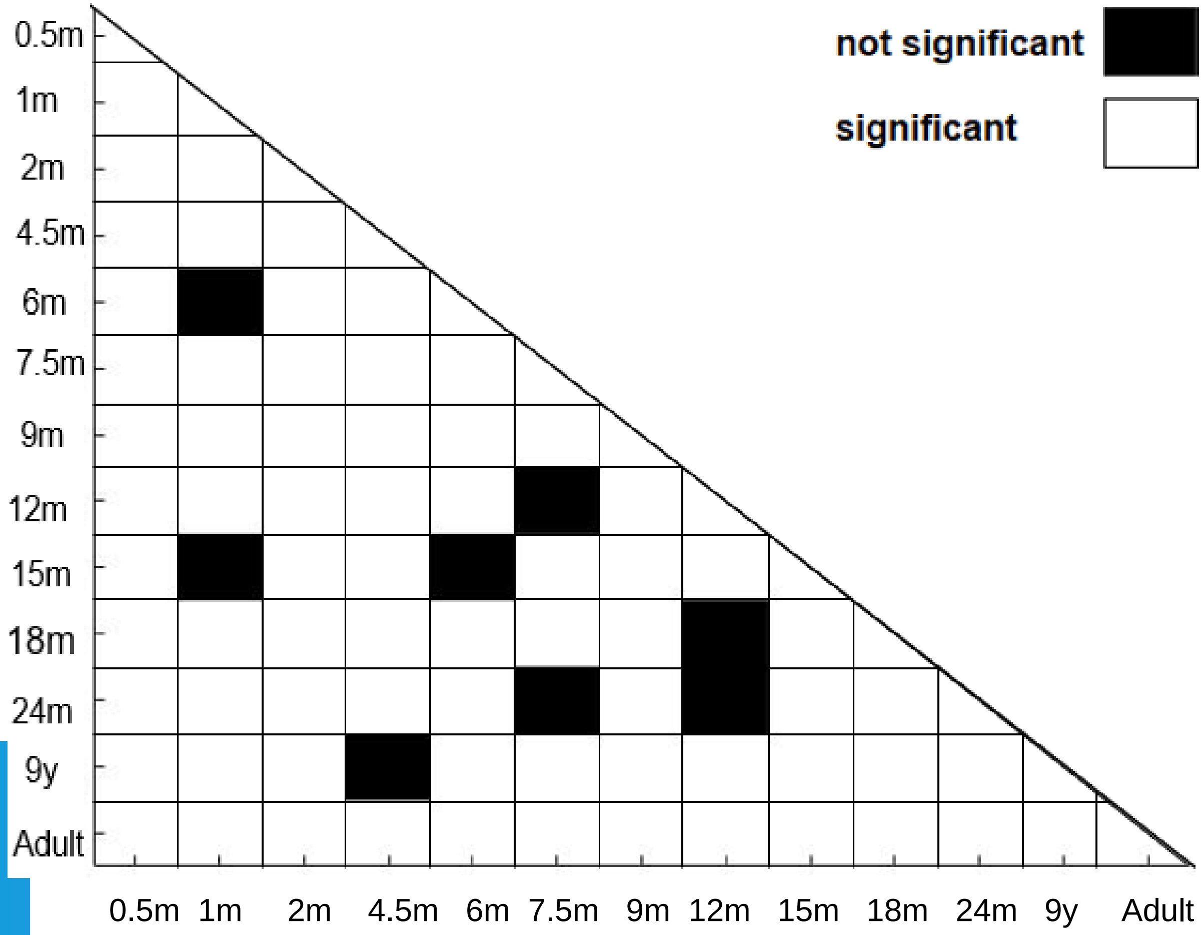

TP (OPMa-mSQUID(Optimized))

ANOVA Table

Source	SS	df	MS	F	Prob>F
Columns	109.05	12	9.08766	180.55	0
Error	9815.51	195013	0.05033		
Total	9924.56	195025			

Multi compare

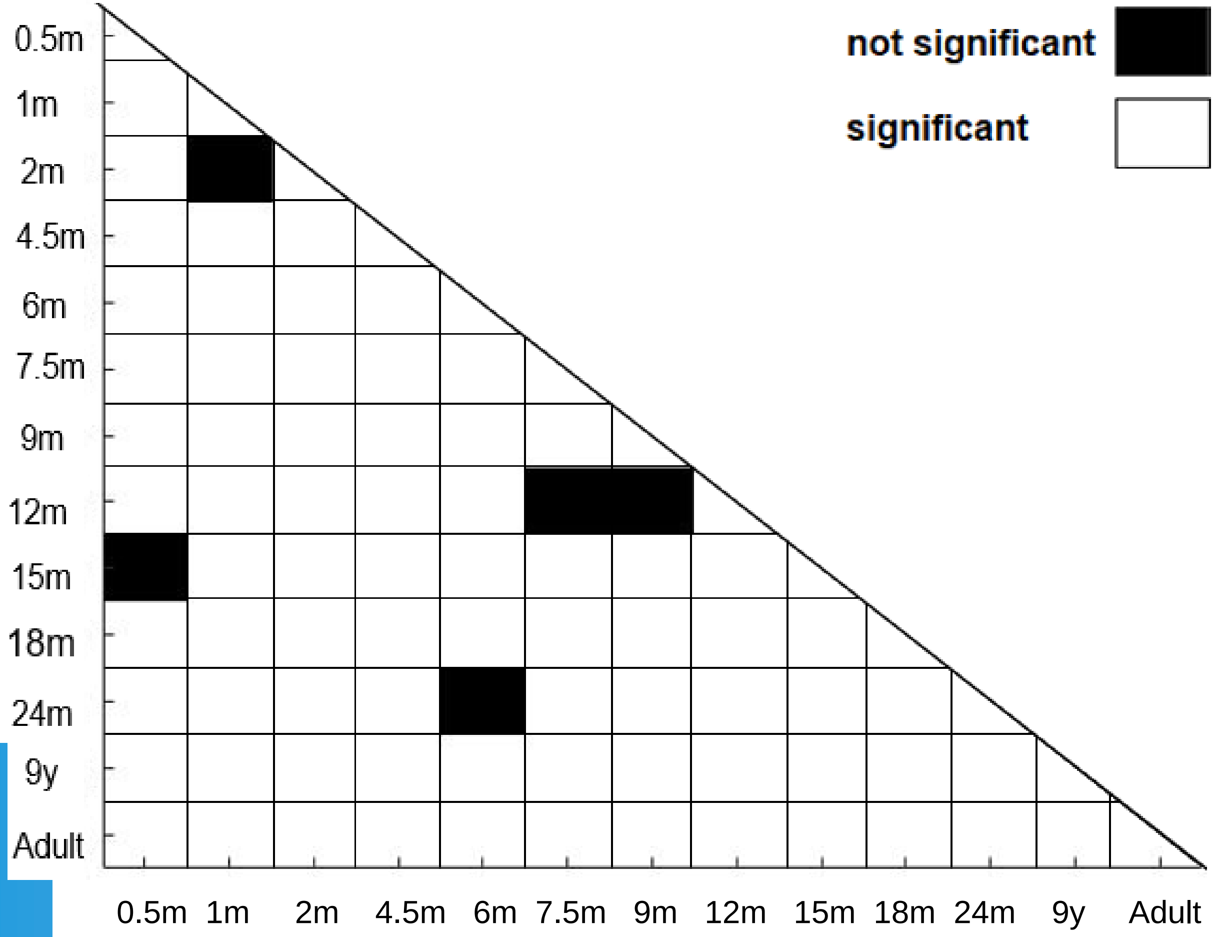

TP (OPMa-mSQUID (Standard))

ANOVA Table

Source	SS	df	MS	F	Prob>F
Columns	203.26	12	16.9385	556.41	0
Error	5936.66	195013	0.0304		
Total	6139.92	195025			

Multi compare

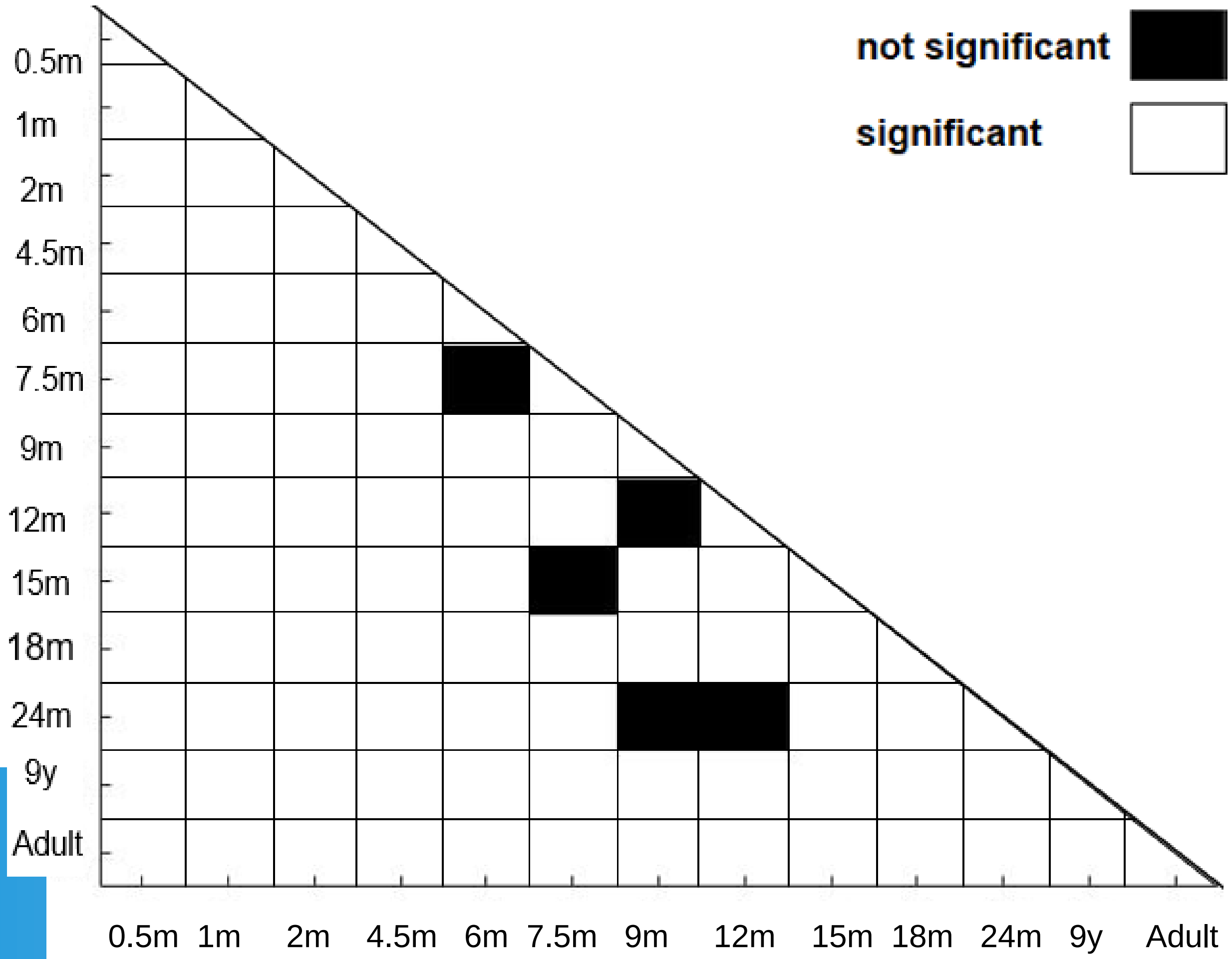

PLE (OPMa-mSQUID(Optimized))

ANOVA Table

Source	SS	df	MS	F	Prob>F
Columns	191.9	12	15.9922	60.63	1.04464e-147
Error	51434.7	195013	0.2638		
Total	51626.6	195025			

Multi compare

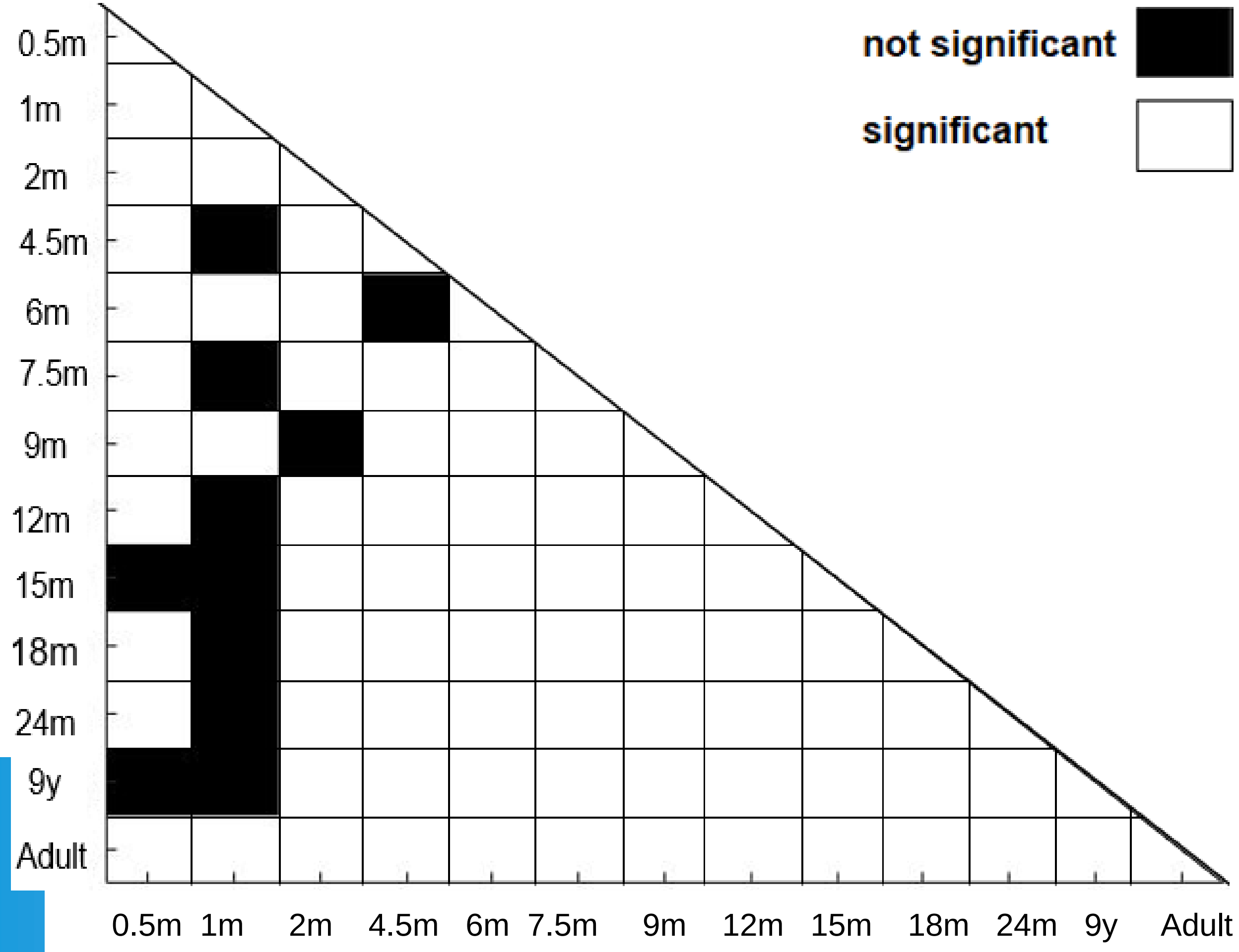

PLE (OPMa-mSQUID (Standard))

ANOVA Table

Source	SS	df	MS	F	Prob>F
Columns	161.5	12	13.4576	35.24	6.70188e-83
Error	74473.6	195013	0.3819		
Total	74635.1	195025			

Multi compare

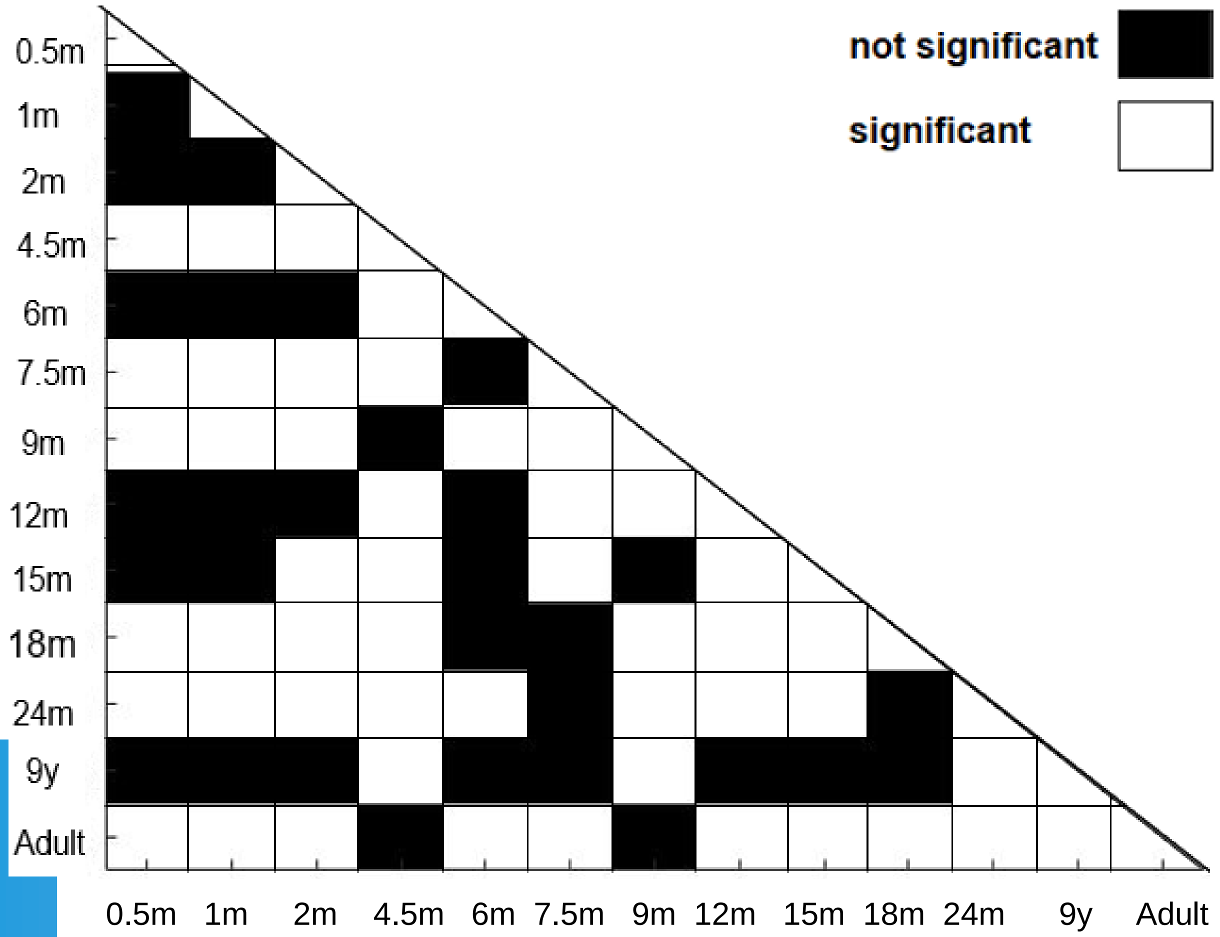

SD (OPMa-mSQUID (Optimized))

ANOVA Table

Source	SS	df	MS	F	Prob>F
Columns	918.5	12	76.5434	660.25	0
Error	22608.1	195013	0.1159		
Total	23526.6	195025			

Multi compare

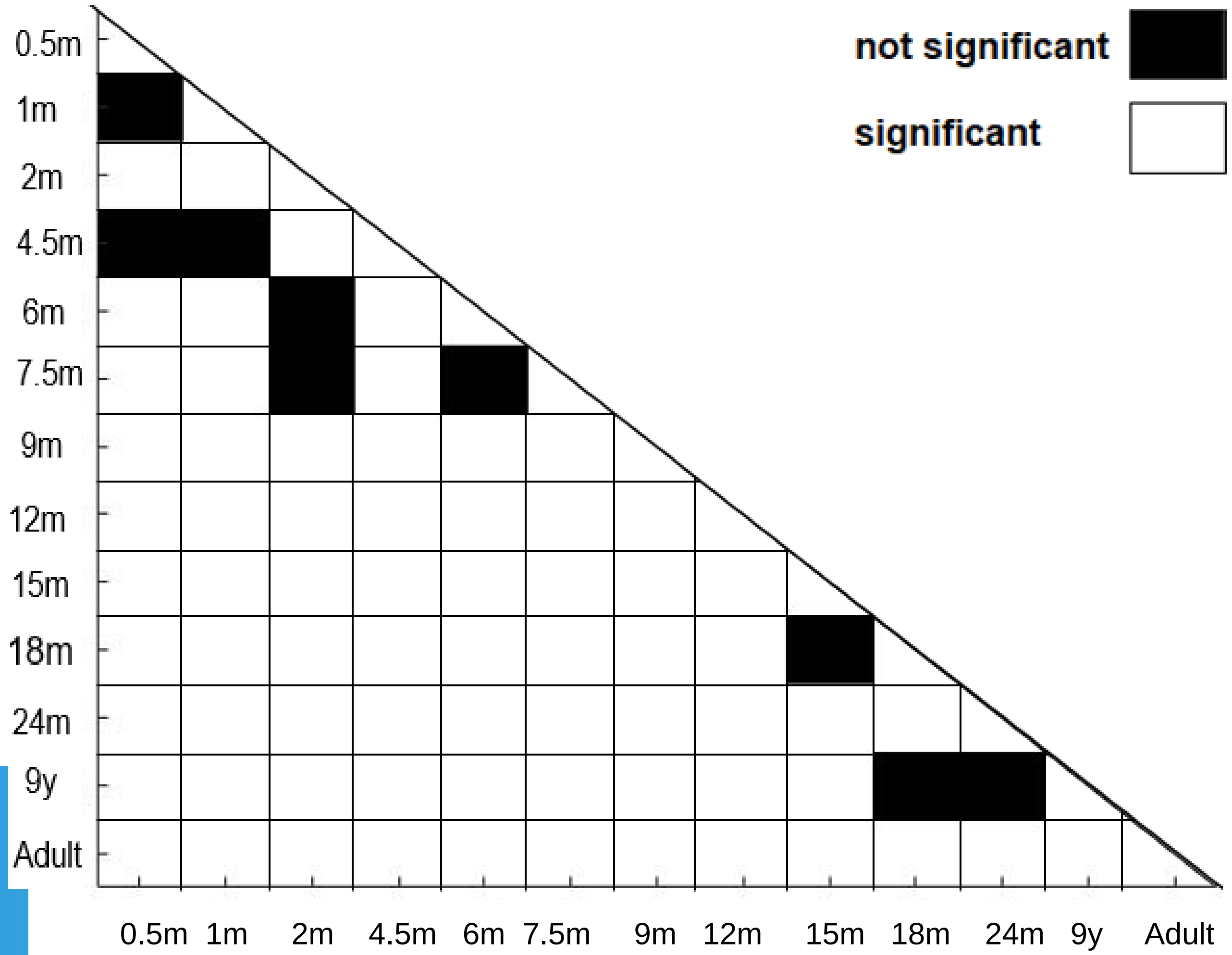

SD (OPMa-mSQUID(Standard))

ANOVA Table

Source	SS	df	MS	F	Prob>F
Columns	952.2	12	79.3502	263.57	0
Error	58710	195013	0.3011		
Total	59662.2	195025			

Multi compare

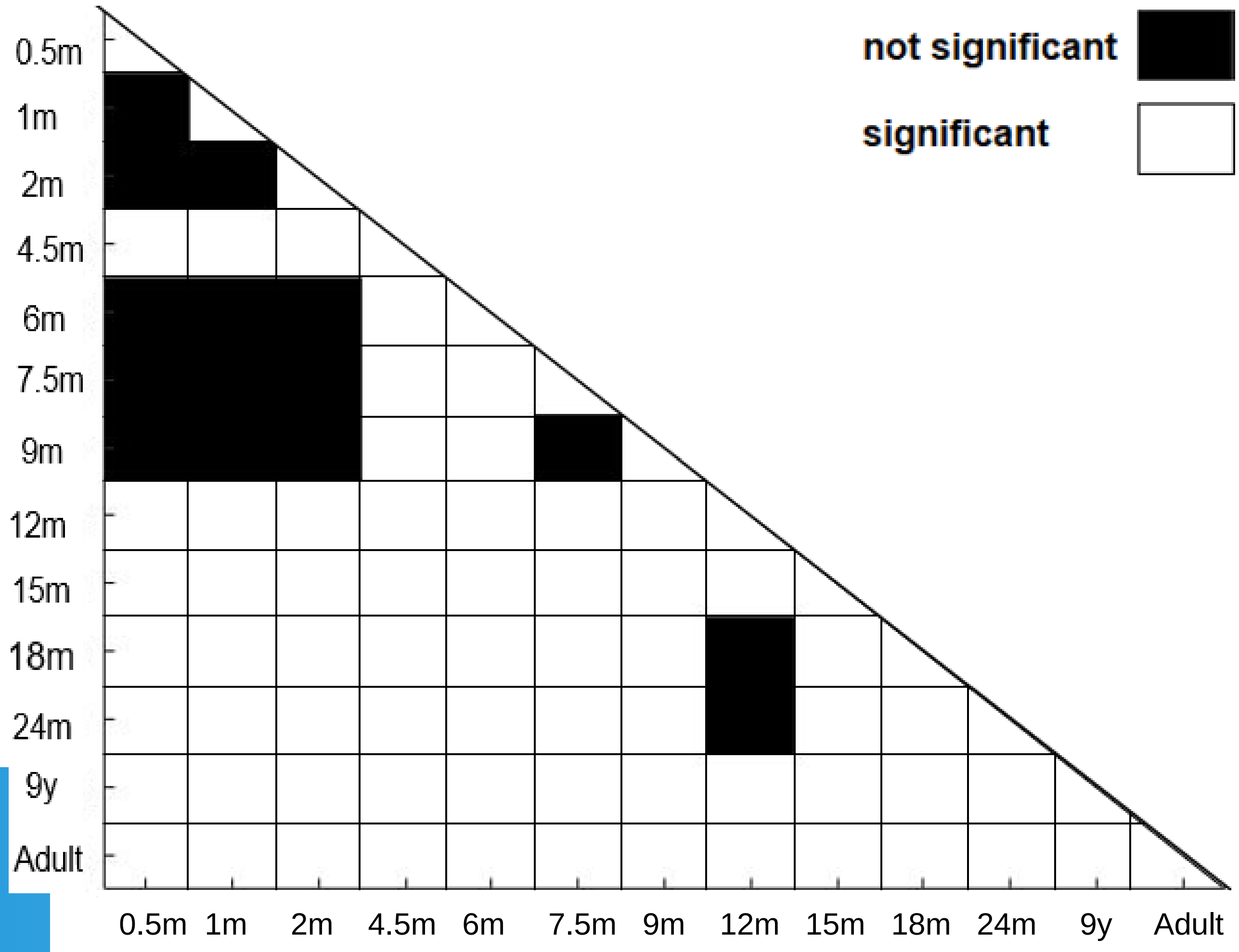

Supplement: Supplementary file 1 [file sensors-22-03093-s001.zip › sensors-1640739-supplementary.pdf]
